# Unlearned adaptive responses to heterospecific referential alarm calls in two bird species from separate evolutionary lineages

**DOI:** 10.1038/s41598-023-47052-5

**Published:** 2023-11-20

**Authors:** Jungmoon Ha, Keesan Lee, Eunjeong Yang, Woojoo Kim, Hokyung Song, Injae Hwang, Larisa Lee-Cruz, Jinseok Park, Jihyeon Song, Chan-ryul Park, Wooshin Lee, Piotr Jablonski, Sang-im Lee

**Affiliations:** 1 Laboratory of Behavioral Ecology and Evolution, School of Biological Science, Seoul National University, Seoul, Republic of Korea; 2 Jeju National University, Jeju-Si, Jeju-Do, Republic of Korea; 3 National Institute of Biological Resources, Seo-Gu, Incheon, Republic of Korea; 4 INRAE, UMR TETIS, Maison de La Télédétection, 500 Rue Jean François Breton, 34090 Montpellier, France; 5 Urban Forests Division, National Institute of Forest Science (NIFoS), Dongdaemun-Gu, Seoul, Republic of Korea; 6 Department of Forest Sciences, CALS, Seoul National University, Seoul, Republic of Korea; 7 Behavioral Ecology Group, Museum and Institute of Zoology, Polish Academy of Sciences, Warsaw, Poland; 8 Laboratory of Integrative Animal Ecology, Department of New Biology, DGIST, Daegu, Republic of Korea

**Keywords:** Evolution, Behavioural ecology

## Abstract

The interspecific responses to alarm signals may be based on unlearned mechanisms but research is often constrained by the difficulties in differentiating between unlearned and learned responses in natural situations. In a field study of two Paridae species, *Parus minor* and *Sittiparus varius*, who originated from a common ancestor 8 million years ago, we found a considerable degree of between-species overlap in acoustic properties of referential snake-alarm calls. Playback of these calls triggered unlearned adaptive fledging behavior in conspecific and heterospecific naive nestlings, suggesting a between-species overlap in the hypothetical unlearned neural templates involved in nestlings’ reactions to alarm calls in both species. This suggests that similar calls and similar unlearned sensitivity might have been present in the common ancestor of the two species, and possibly in the ancestor of the whole family Paridae that originated 10–15 million years ago in Asian regions rich in snakes.

## Introduction

It is known that many animals can learn and generalize the meaning of conspecific and heterospecific alarm calls. With repeated exposure to alarm calls and simultaneous perception of the predator's presence, either directly or through observing other individuals' behavioral responses^1–3^, they become more adept at responding appropriately to such calls (whether conspecific or heterospecific). Studies on learned responses to heterospecific alarm calls have predominantly focused on sympatric species^4–6^, where natural opportunities for cross-species learning exist. However, the potential role of unlearned sensitivity (innate template) to conspecific alarm calls or the role of general sensitivity of the neurosensory system to intrinsic properties of alarm calls may be difficult to conclusively determine in interspecific response studies^7–9^. This challenge stems from experiments being primarily conducted on adult individuals, who likely acquired associations between alarm calls and predator (danger) types through individual learning during their lifetime^10,11^. Adult birds that are outside the nest and are capable of hearing alarm calls and observing predators, not only develop a hypothetical template to acoustically recognize specific alarm calls but also actively seek patterns specific to the predators that the alarm calls concern. Consequently, distinguishing the effects of prior experiences from those arising by genetic and developmental processes, which do not involve learning, becomes intricate in typical natural situations^7,12–14^. Furthermore, investigating unlearned/innate interspecific reactions to heterospecific alarm calls require the use of naïve individuals—those without prior exposure to alarm calls and their associated situations—a task that can be difficult to achieve in typical natural settings. As a consequence of these challenges, conducting studies on unlearned/innate interspecific reactions to conspecific and heterospecific alarm calls in natural habitats is relatively rare.

In this context, the recently described behavioral response of Oriental tit (*Parus minor*; also known as the Japanese tit, as per IOC standards) nestlings to parental referential snake alarm calls, known as the ‘jar’ calls^15–17^, presents a unique opportunity to explore unlearned responses to alarm calls given by adult birds near nest cavities with nestlings. Similar to many other species in the Paridae family^18^, the Oriental tit breeds in tree cavities or nest boxes. Snakes pose a threat to nestlings within nest cavities, and Oriental tit parents use referential alarm calls in response to snake approaches to nests. These calls consist of repetitions of a single syllable type falling under the broader classical category of ‘LF/churr/rattle/D/jar’ syllables (hereafter referred to as ‘churr’) in Paridae^17,19–22^, and were specifically named as ‘jar’ calls by Suzuki^15–17^. Currently, it remains unknown whether these calls are acquired through unlearned or learned mechanisms, but there is sufficient evidence supporting the idea that that reactions of nestlings to those calls are innate (not acquired through learning, hence, unlearned). Nestlings inside a nest cavity can hear alarm calls from birds (including heterospecifics) outside the cavity, but they cannot see the predator or their parents. Consequently, these nestlings cannot learn the associations between specific alarm calls and the presence of a particular type of predators outside their nests or the behavior of adult birds near the nest. Therefore, the reactions of nestlings to alarm calls given by their parents outside the nest cavity must be based on innate developmental processes rather than on learning. Previous studies on nestlings of cavity-nesting bird species have reported behavioral reactions to various alarm vocalizations of adults calling from outside the nest. The nestlings often become quiet and crouch in the nest, a response common across various bird taxa^23^. However, when Oriental tit nestlings reach an older age and become physically capable of jumping out of the nest, even before the typical fledging time (they may not yet be able to fly), they exhibit an unlearned behavioral response specific to the referential snake alarm calls: they jump out of the nest^15–17^. They do not exhibit this behavior that in response to parental alarm calls induced by corvid or mammal predators^15,17^. This observed behavior strongly suggests a match between the nestlings’ unlearned, developmentally acquired auditory sensitivity to the snake alarm calls and the acoustic features of those calls. This situation differs from the response of ready-to-fledge nestlings to parental recruitment calls known in birds to induce fledging in non-threatening situations. Considering their context, these recruitment calls are likely of a non-referential nature, and they have not been systematically described in Paridae (it is possible that they also contain syllables of the general ‘churr’ type).

Since the sensitivity of Oriental tit nestlings to the snake alarm calls and their jump-out response to these calls are likely based on an unlearned template (that develops with nestlings' age), it may, in principle, be possible to trace the evolutionary history of those innate characteristics. Hence, given the recently determined phylogeny of the family Paridae^24^, this group presents unique research opportunities to gain insights into the evolution of unlearned templates and behavioral responses to referential alarm calls. However, currently, only the Oriental tit has been studied in this regard. Thus, as the first step to provide a background for generating specific hypotheses for future phylogeny-wide comparisons among multiple Paridae species, we chose to compare the alarm calls and nestlings' responses to the snake alarm calls in the Oriental tit with those of the Varied tit (*Sittiparus varius*). These two species are located on separate branches of the Paridae phylogeny, representing two main evolutionary lineages^24^.

The two species are sympatric and share the same habitat. Habitat physical properties are likely to similarly affect the basic acoustic properties of all alarm calls in response to a diversity of threats in both species, and the two species are exposed to the same predators, including snakes^25^. Hence, in addition to the effects of common ancestry, the call structure may be relatively similarly affected by habitat-specific factors in these species. As snakes pose a similar threat to the Varied tit nestlings and to the Oriental tit nestlings^25^, we expected that the Varied tit might also possess parental snake-specific referential alarm calls and fledging responses similar to those of the Oriental tit. Therefore, we aimed to determine whether this is the case and to what extent the acoustic properties of the Varied tit's alarm vocalizations in response to various types of predators, including snakes, overlap with or differ from those of the already studied Oriental tit alarm calls. Considering that both species are exposed to the same predators, including snakes^25^, we further investigated whether Varied tit nestlings respond to their parents’ snake alarm calls in a manner similar to Oriental tit nestlings. Finally, based on the detected apparently higher overlap in the acoustic properties of the snake alarm calls between the two species compared to the overlap between the vocalizations to non-snake predators, we aimed to test if this overlap is sufficient to induce the nestlings’ jump-out reaction in response to heterospecific snake alarm calls in both the Oriental and the Varied tit.

The overarching aim of our research is to contribute to understanding of alarm call evolution in the family Paridae. The initial focus on the two species offers a solid background for formulating specific hypotheses about both ultimate and proximate mechanisms involved in the evolution of alarm calls within the entire Paridae family. By investigating the alarm call properties and nestling responses in these two species, which come from separate lineages within the Paridae phylogeny, we provide a foundation for the future quantitative comparative analyses across the full phylogeny. This will allow us to reconstruct evolutionary history of alarm calls, particularly those related to snakes, and to understand the mechanisms involved in the evolution of these calls. Such mechanisms include the effects of common ancestry and the effect of habitat, which can be evaluated in the future phylogeny-wide analyses involving multiple species.

## Results

### Auditory responses induced by different predators

**Figure 1:**
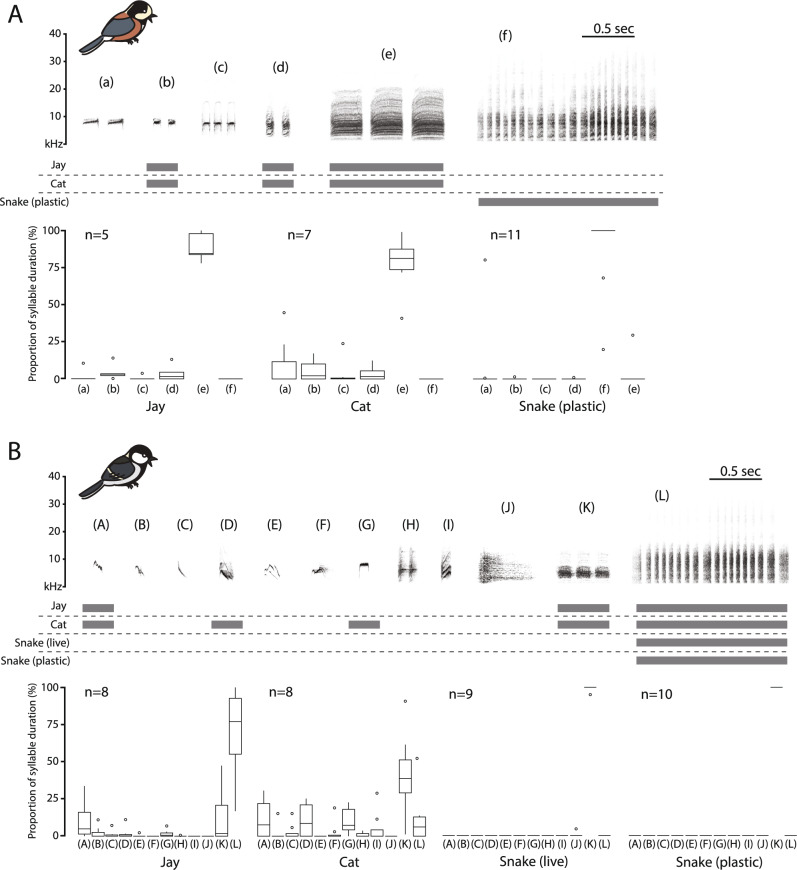
Examples of typical sonograms of syllable types recorded in parental alarm calls in response to three predators: jays, cats, and snakes by the Varied tit (**A**) and the Oriental tit (**B**). Grey boxes under each syllable type indicate that they were observed in respective experimental treatments, with the median proportion of each syllable type calculated across multiple samples for each treatment exceeding 1%. Proportions were determined by aggregating the duration of all syllables in each syllable type in a recording and dividing the value by the total duration of all syllables within that recording. Additionally, boxplots illustrating the proportions of syllable types are provided beneath the sonogram of syllables for each species. The bird images of the both study species were drawn by Jungmoon Ha, the first author of the paper.

Visual inspection of all sonograms of auditory responses obtained during the experimental presentations of the three predators (jays, cats, and snakes) provides insights into the differences among alarm calls triggered by these predators, representing three predator types: birds capable of capturing fledglings, mammals capable of capturing both adults and fledglings, and snakes that enter nest cavities and destroy entire broods. The two species used different syllable types, and examples of vocalizations to different predators are available in Supplementary Videos V1, V2, V3, and Supplementary Audio files S1, S2. We identified 6 syllable types in Varied tit vocalizations and 12 syllable types in Oriental tit vocalizations (Fig. 1). Within each species, vocalizations triggered by the presentation of the three predator types differed in the types of syllables used and in the frequency of use of these syllable types. The parental responses of both tit species to a jay or a cat were relatively complex, incorporating 3–9 syllable types, each with different acoustic properties between the two species (Fig. 1; Supplementary Videos V1, V2). However, some of these syllables were not frequently and consistently present in every response. The typical syllable types used in responses to a jay were syllables ‘b’, ‘d’, ‘e’, and ‘A’, ‘K’, ‘L’ in the Varied tit and the Oriental tit, respectively. For responses to the cat model, the typical syllable types were ‘b’, ‘d’, ‘e’, and ‘A’, ‘D’, ‘G’, ‘K’, ‘L’ in the Varied tit and the Oriental tit, respectively (Fig. 1).

In contrast, the vocal responses to snakes near nests were less complex, consisting almost entirely of one repeated syllable type in phrases: syllable ‘f’ in the Varied tit and syllable ‘L’ in the Oriental tit (Supplementary Video 3; Supplementary Audios S1, S2). Among the syllable types of the Varied tit, we found that the majority of syllables produced in response to the snake were the ‘f’ syllables, with the proportions of ‘f’ syllables induced by the other two predators approaching near zero (Fig. 1). While the proportion of ‘L’ syllables was also high in the recordings from Oriental tits during jay and cat presentations, these recordings additionally contained several other syllables, in contrast to recordings from snake presentation tests.

**Figure 2:**
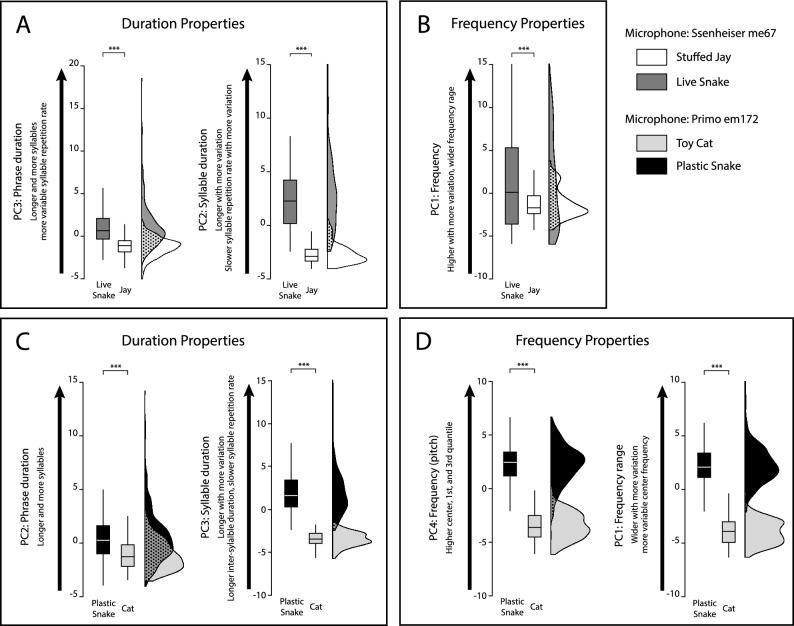
Acoustic properties of the ‘L’ syllables in Oriental tit responses to different predators. Boxplots and raincloud plots display varimax-rotated principal components for ‘L’ phrases of the Oriental tit, comparing snake *vs*. jay (**A**,**B**), and snake *vs.* cat (**C**,**D**) responses. The principal components are grouped into those representing duration properties (**A**,**C**) and those representing frequency properties (**B**,**D**). The proportion of variance explained by these components vary from 17 to 24% (details in Supplementary Tables S1, S2). The dotted area in the raincloud plots indicates overlapping areas between frequency distributions of values between treatments, and the linear overlap of the bases of these distributions illustrates the ranges of values that overlap between treatments. Please note that these plots do not account for the random effect of nest ID included in the statistical analysis. For a comprehensive overview of the statistical analysis results, please refer to Supplementary Tables S1 and S2. Additionally, Supplementary Fig. S2 presents boxplots depicting the original duration and frequency variables contributing to the principal components.

As a simple visual inspection of sonograms did not allow for reliable differentiation between various ‘L’ type syllables, we conducted a statistical analysis to determine if and how the ‘L’ elements differ among the Oriental tit’s responses to the three different predators in terms of duration/timing properties and frequency properties. The duration variables for each phrase comprised the Number of Syllables, Phrase duration, Average Syllable Duration, Standard Deviation of Syllable Duration, Average Calling Rate, Standard Deviation of Calling Rate, and Average Inter-syllable Interval. The frequency variables comprised the Average Center Frequency of Syllables within a Phrase; Standard Deviation of Center Frequency of Syllables within a Phrase; Average 1st Quartile of Frequency Distribution in a Syllable within a Phrase, Average 3rd Quartile of Frequency Distribution in a Syllable within a Phrase, Average Interquartile Frequency Range in a Syllable within a Phrase (see details in Supplementary Table S6, Supplementary Fig. S2). By using principal component analysis, we extracted several composite variables, each explaining 17–32% of the variability, and representing separate aspects of the acoustic properties: phrase duration, syllable duration, frequency pitch and range (or one variable representing variability in both pitch and range; see Fig. 2 and Supplementary Tables S1, S2 for additional details). We found statistically significant differences in all principal components representing frequency and duration characteristics among the ‘L’ syllables in response to the three different predators (snake vs. jay comparison; PC1: p-value < 0.001, PC2: p-value < 0.001, PC3: p-value < 0.001, snake vs. cat comparison; PC1: p-value < 0.001, PC2: p-value < 0.001, PC3: p-value < 0.001, PC4: p-value < 0.001; see Supplementary Tables S1, S2 for details). The results also exhibited some degree of overlap, particularly in terms of frequency features in the snake-jay comparison and phrase duration characteristics in the snake-cat comparison (Fig. 2; Tables 1, 2).

**Table 1:** Variance component analysis results for principal components of ‘L’ phrases in Oriental tit responses to live snake and jay presentations. This table represents the results of Variance Component Analysis (conducted using the ‘VCA’ function in the R package ‘VCA’) for varimax-rotated principal components derived from 13 characteristics of ‘L’ phrases (PC1, PC2, PC3; see Supplementary Table S1). The analysis assesses the proportion of variance in each principal component explained by the predator type during live snake and jay presentation experiments, recorded with a Sennheiser me67 microphone (see Table 4 in Methods). The smaller proportion of total variance explained by the predator type (indicated in bold underlined font) indicates the larger degree of overlap in acoustic properties between calls given in response to the two different predators. The error can be interpreted as a within-trial variation of the principal component. This table also corresponds to Figs. 2A,B.

Principal component	Levels of variance		df	Variance	% of total variance	SD
**PC1: Frequency** *(pitch, range, variability)*	Total	17.79		19.23	100	4.38
	**Predator**	1		0.97	**5.04**	0.98
	Predator/nest	15		15.13	78.68	3.89
	Error	446		3.12	16.26	1.76
**PC2: Syllable** (*syllable duration & its variability, call rate & its variability*)	Total	1.91		18.99	100	4.35
	**Predator**	1		13.38	**70.48**	3.65
	Predator/nest	15		2.21	11.67	1.48
	Error	446		3.38	17.84	1.84
**PC3: Phrase** (*phrase duration, syllable number, call rate variability*)	Total	6.75		7.83	100	2.79
	**Predator**	1		2.76	**35.26**	1.66
	Predator/nest	15		1.55	19.79	1.24
	Error	446		3.52	44.93	1.87

**Table 2:** Variance component analysis results for principal components of ‘L’ phrases in Oriental tit responses to plastic snake and cat presentations. This table represents the results of Variance Component Analysis (conducted using the ‘VCA’ function in the R package ‘VCA’) for varimax-rotated principal components derived from 13 characteristics of ‘L’ phrases (PC1, PC2, PC3, PC4; see Supplementary Table S2). The analysis assesses the proportion of variance in each principal component explained by the predator type during plastic snake and cat presentation experiments, recorded with a Primo EM172 microphone (see Table 4 in Methods). The smaller proportion of total variance explained by the predator type (indicated in bold underlined font) indicates the larger degree of overlap in acoustic properties between calls given in response to the two different predators. The error can be interpreted as a within-trial variation of the principal component. This table also corresponds to Figs. 2C,D.

Principal component	Levels of variance	df	Variance	% of total variance	SD
**PC1: Frequency** (*range & its variability*)	Total	1.27	21.76	100	4.66
	**Predator**	1	19.18	**88.12**	4.37
	Predator/nest	16	0.53	2.43	0.72
	Error	500	2.05	9.43	1.43
**PC2: Phrase** (*phrase duration, syllable number, call rate variability*)	Total	17.12	6.49	100	2.54
	**Predator**	1	1.34	**20.62**	1.15
	Predator/nest	16	1.11	17.22	1.05
	Error	500	4.03	62.14	2.00
**PC3: Syllable** (*syllable duration & its variability, intervals, call rate*)	Total	1.84	20.79	100	4.56
	**Predator**	1	15.14	**72.83**	3.89
	Predator/nest	16	0.99	4.76	0.99
	Error	500	4.65	22.39	2.15
**PC4: Frequency pitch** (*pitch*)	Total	1.36	18.78	100	4.33
	**Predator**	1	15.85	**84.37**	3.98
	Predator/nest	16	1.36	7.24	1.16
	Error	500	1.57	8.38	1.25

### Comparison of snake alarm calls between the Varied tit and the Oriental tit

The snake alarm call was composed of one type of syllable in both species: type ‘f’ in the Varied tit and type ‘L’ in the Oriental tit (Fig. 1). Notably, the ‘L’ phrases in the snake alarm calls of the Oriental tit exhibited acoustic properties distinct from the ‘f’ phrases of the Varied tit (PC1: p-value < 0.001, PC2: p-value < 0.001, PC3: p-value < 0.001, PC4: p-value < 0.001; please refer Supplementary Table S3).

**Figure 3:**
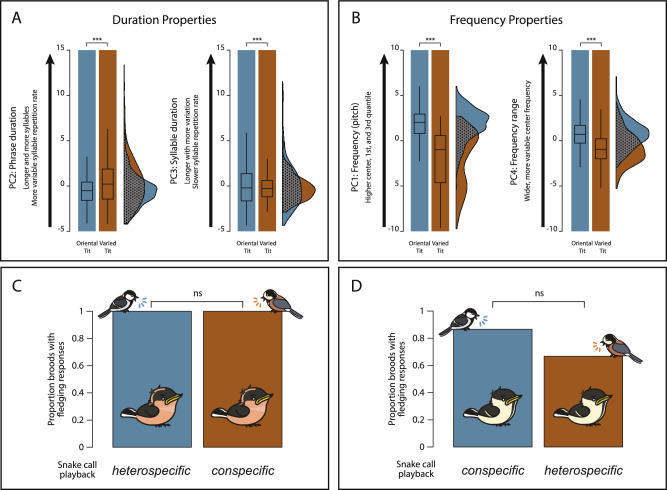
Acoustic properties of snake alarm calls in the Oriental tit and the Varied tit, and the responses they trigger in the nestlings of these species. (**A**,**B**) Boxplots and raincloud plots illustrating varimax-rotated principal components for comparing ‘L’ phrases of the Oriental tit and ‘f’ phrases of the Varied tit in response to the plastic snake. The dotted areas in the raincloud plots for the respective components show the overlaps between the two data distributions. The linear overlap of the bases of these distributions illustrates the ranges of values that overlap between treatments. Please note that these plots do not account for the random effect of nest ID included in the statistical model. For a comprehensive overview of the statistical analysis results, please refer to Supplementary Table S3. Additionally, Supplementary Fig. S3 presents boxplots depicting the original variables. (**C**,**D**) Proportion of Oriental tit and Varied tit broods with observed fledging response of nestlings triggered by conspecific or heterospecific playback of "snake calls", relative to the total number of broods tested. Proportions were separately calculated for tests involving conspecific and heterospecific calls for each nestling species. The bird images of the both study species were drawn by Jungmoon Ha, the first author of the paper.

Although statistically different, there was a relatively extensive degree of overlap in specific acoustic properties (represented by the separate Principal Components) between the two species (Fig. 3A,B; Table 3) compared to the analogous overlaps in the Oriental tit's ‘L’ phrases for the snake vs. jay and snake vs. cat comparisons (Fig. 2; Tables 1, 2). These acoustic overlaps of snake alarm calls between the two species were primarily related to three composite variables: the “Phrase duration” (PC2, representing mostly phrase duration, number of syllables, and syllable repetition variability; Fig. 3A), the “Syllable duration” (PC3, representing mostly syllable duration and syllable repetition rate; Fig. 3A), and “Frequency range” (PC4, representing the width of frequency range and variability of center frequency within a phrase; Fig. 3B). These overlaps correspond to approximately 5%, 0%, and 30% of the variance explained by species for “Phrase Duration” (PC2), “Syllable Duration” (PC3) and “Frequency Range” (PC4), respectively (Table 3), indicating a considerable overlap between species. A similar variance component analysis in the comparisons between the Oriental tit calls in snake and cat treatments revealed that predator type explained about 21%, 73%, and 88% of the variance in “Phrase Duration” (PC2), “Syllable Duration” (PC3), and “Frequency Range” (PC1), respectively (Table 2). This suggests a relatively small overlap between the Oriental tit calls in response to the two predator types compared to the overlap in snake alarm calls between the two tit species, particularly for syllable duration and frequency range/variation properties. In a similar variance component analysis for the snake–jay comparison, syllable duration properties also showed a low degree of overlap between the predator types, with 70% of the variance explained by predator type (Table 1). Frequency properties in this analysis were not divided into separate pitch and range components, and the “frequency properties” (PC1) were similar between snake and jay, with approximately 1% of the variance explained by predator type (Table 1).

**Table 3:** Variance component analysis results for principal components of ‘L’ phrases in Oriental tit and Varied tit responses to plastic snake. This table represents the results of Variance Component Analysis (conducted using the ‘VCA’ function in the R package ‘VCA’) for varimax-rotated principal components derived from 13 characteristics of ‘L’ phrases (PC1, PC2, PC3, PC4; see Supplementary Table S3). The analysis assesses the proportion of variance in each principal component explained by the species during plastic snake presentation experiments for the two species, recorded with a Primo EM172 microphone (see Table 4 in Methods). The smaller proportion of total variance explained by the species (indicated in bold underlined font) indicates the larger degree of overlap in acoustic properties between calls of the two species given in response to the plastic snake. The error can be interpreted as a within-trial variation of the principal component. This table corresponds to Figs. 3A & 3B.

Principal Component	Levels of variance	df	Variance	% to total variance	SD
**PC1: Frequency pitch** **(24%)** (*pitch*)	Total	3.54	12.84	100	3.58
	**Species**	1	6.30	**49.08**	2.51
	Species/nest	19	4.05	31.60	2.01
	Error	589	2.47	19.30	1.57
**PC2** (*phrase duration, syllable number, call rate variability*)	Total	127.49	5.96	100	2.44
	**Species**	1	0.30	**5.15**	0.55
	Species/nest	19	1.00	16.79	1.00
	Error	589	4.65	78.05	2.15
**PC3** (*syllable duration & its variability, call rate*)	Total	254.54	4.95	100	2.22
	**Species**	1	0	**0**	0
	Species/nest	19	1.02	20.63	1.01
	Error	589	3.93	79.36	1.98
**PC4** (*range & pitch variability*)	Total	9.25	4.00	100	2.00
	**Species**	1	1.19	**29.85**	1.09
	Species/nest	19	0.88	22.01	0.93
	Error	589	1.92	48.13	1.38

Finally, the examination of seven duration property variables and six frequency property variables in Supplementary Fig. S2 and S3 suggests six variables that exhibit relatively little overlap in ranges in snake-jay and snake-cat comparisons (panels marked with an asterisk in Supplementary Fig. S2) and relatively extensive range overlap in the Oriental Tit—Varied Tit snake alarm calls comparisons (panels marked with an asterisk in Supplementary Fig. S3): Average Syllable Duration, Average Calling Rate, Standard Deviation of Calling Rate, Standard Deviation of Center Frequency of Syllables within a Phrase, Average 1st Quartile of Frequency Distribution within a Syllable in a Phrase, Standard Deviation of Interquartile Range of Frequency of Syllables in a Phrase.

### Fledging of Oriental tit and Varied tit nestlings in response to snake alarm calls

Given the relative overlap in some acoustic properties between the snake alarm calls of the two species, we asked if nestlings of the two species would jump out of the nest in response to both conspecific and heterospecific snake alarm calls. The Varied tit nestlings responded to both conspecific (n = 10) and heterospecific (n = 10) snake-alarm calls by exhibiting a jump-out behavior from the nest boxes in 100% of the tests (Fig. 3C,D; Supplementary Table S4). Furthermore, our observations in this population confirmed that the Oriental tit nestlings also responded to conspecific snake-alarm calls by jumping out of the nest box in a majority, although not all, of the tests (in 13 out of 15 tests). This frequency of fledging responses did not differ statistically from the responses to heterospecific snake-alarm calls by the Oriental tit (Fisher's exact test, odds ratio (95% CI) = 0.32 (0.02–2.47), p-value = 0.389; Mixed effects logistic regression, odds ratio (95% CI) = 0.30 (0.04–1.93), p-value = 0.219).

## Discussion

We observed limited similarities in the vocalizations directed towards cats and jays between the two species, primarily because they use different syllables in those alarm vocalizations. This indicates that, despite sharing a common ancestor, and despite coexisting and communicating acoustically in the same habitat with specific physical characteristics that could influence sound propagation, the vocal alarm responses to different predators are species-specific. Furthermore, the utilization of various syllable types in response to non-snake predators is consistent with previous research on Paridae species^20,26–28^.

The Varied tit parents employed a unique, species-specific snake alarm call, prompting their nestlings to jump out of the nest when they were able to fledge. This pattern of communication, involving unlearned responses to snake alarm calls, closely parallels the behavior observed in the Oriental tit^15,17,26^. Moreover, we observed substantial acoustic overlap between some properties of the Varied tit’s snake alarm calls and those of the Oriental tit, in contrast to calls directed at other predators like jays and cats. We propose a hypothesis suggesting that this acoustic overlap is linked to the nestlings' responses to heterospecific snake alarm calls observed in our experiments. We postulate that at least some features of this overlap match the putative hypothetical innate template representing key acoustic features that trigger the fledging response in older nestlings. In contrast to the relatively complex auditory responses towards non-snake predators, we consistently identified only one atonal syllable type (albeit statistically distinct between the two species) in each snake alarm call. This observation is consistent with the idea that the hypothetical innate template encompasses simple acoustic features. However, since we did not conduct playback experiments to test if old nestlings of the Varied Tit indeed do not fledge in response to non-snake alarm calls from conspecifics, future studies should investigate this issue for clarification.

Magrath^2^ proposed that acoustic similarities between functionally equivalent alarm calls of different species could facilitate the evolution of unlearned or innate interspecific behavioral responses to those alarm calls. While we observed some statistically significant differences in the acoustic responses between the two Paridae species, the fundamental principles of generalization in signal detection, recognition, and behavioral responses to signals might have led to interspecific responses to alarm calls with shared features^29–31^. Such a signal generalization in acoustic recognition may occur when the cost of ignoring a signal outweighs the cost of responding to a misclassified stimulus by mistake^32^. This mechanism enables appropriate responses to the key acoustic features of conspecific alarm calls despite their variability due to various factors^33,34^. Previous studies on bird alarm communication^13,30,35,36^ have suggested that naive individuals, without any prior experience, can respond to heterospecific alarm calls if these calls share key acoustic features with their conspecific calls. We hypothesize that such a generalization might have occurred in our study system of the two Paridae species.

What might be the shared key acoustic features of snake alarm calls between the two species? The "snake calls" of the Oriental tit and the Varied tit consisted of repeated atonal syllables within a phrase, characterized by a wide frequency band (the ‘jar’ version of the ’L’ syllable in the Oriental tit and the ‘f’ syllable in the Varied tit). Given the acoustic similarity between snake calls in both species, it can be inferred that these calls share a higher degree of similarity in syllable duration/rate properties, as well as frequency range and variability within a phrase. Based on these observed similarities, we propose a hypothesis that the putative unlearned developmentally acquired template responsible for triggering the nestlings' jump-out response also involves sensitivity to these shared acoustic features. Furthermore, these shared acoustic features distinguish species-specific snake alarm calls from non-snake alarm calls that did not elicit fledging responses in conspecific nestlings^15,17^. Future experiments and analyses across multiple species of Paridae should focus on identifying the specific variables shared within the ‘churr’ calls that have the potential to trigger nestling fledging and differentiate them from other ‘churr’ calls that lack this propensity.

Several studies on birds have identified various acoustic variables that may serve as the key features that are crucial for inducing responses to heterospecific alarm calls. For instance, in the Maluridae family, multiple species appear to recognize heterospecific aerial alarm calls as long as the peak frequency and frequency modulation are similar to their conspecific calls^29^. Similarly, in apostlebirds, the dominant frequency, rather than the broad bandwidth of the alarm call, was found to be critical for eliciting an interspecific mobbing response^7^. Other examples of interspecific recognition include the European Great tit (*Parus major*), which can discern the meaning of alarm calls from the Black-capped Chickadee (*Poecile atricapillus*) based on the number of specific syllables (‘D’ notes) in a phrase, conveying a sense of urgency^14^. Additionally, similar interspecific transfer of urgency information was observed between the White-browed Scrubwren (*Sericornis frontalis*) and the Superb Fairy-wren (*Malurus cyaneus*)^37^. Unlike our study, most of these examples involve alarm calls likely influenced by learning or where at least the role of learning cannot be excluded. Therefore, our study contributes to understanding and guides future directions in studies of mechanisms responsible for unlearned heterospecific responses to referential alarm calls.

**Figure 4:**
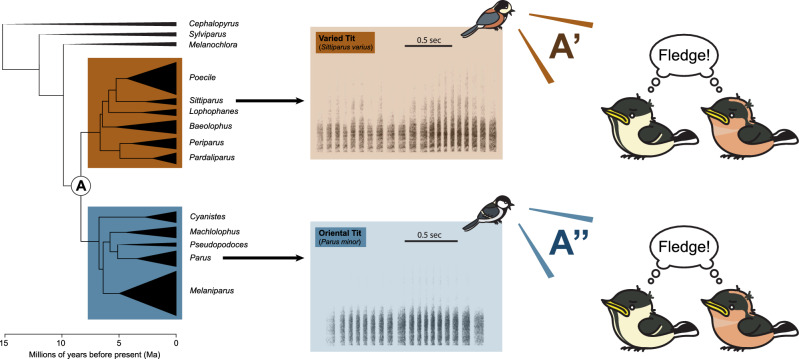
Summary and significance of the results for future research. A simplified phylogenetic tree of the Paridae family, depicting the positions of the Oriental Tit and the Varied Tit, along with representative sonograms of their "snake calls". The subfigure on the right illustrates the observed fledging behavior of nestlings from these two subject species, triggered by playback of conspecific and heterospecific "snake calls." This phylogenetic tree is based on the work of Johansson et al. (2018)^24^. The bird images of the both study species were drawn by Jungmoon Ha, the first author of the paper.

The observed overlap in call properties and the receiver's sensitivity is consistent with the general mechanisms underlying the evolution of predator-specific alarm signals. As the anti-predatory signals in prey animals often show specificity to the predator type or taxon^38–41^, natural selection may have driven the evolution of alarm calls and anti-predatory behaviors that are specific to predatory snakes, sharing similarities across multiple prey species. Given that acoustic snake alarm signals that match the innate sensitivity of nestlings may bring crucial fitness benefits in habitats with high risk of nest predation by snakes, it is plausible that natural selection prompted parental snake alarm calls to adapt to nestling sensitivity by incorporating fundamental acoustic elements that trigger fledging. Hence, we hypothesize that the putative shared pre-existing template ("pre-existing sensitivity" hypothesis) may have contributed to the natural selection for similarities between species-specific snake calls and the adaptive fledging response in nestlings triggered by alarm calls referentially associated with the predator type that poses a threat to nestlings inside nest cavities, but not the alarm calls to other predators. If this scenario holds true, it would imply that over 8 million years of evolutionary history along two separate branches of the Paridae's phylogeny (Fig. 4), the template and call of the two species underwent relatively small changes, resulting in conspecific as well as heterospecific responses by nestlings from the two species. However, the extent to which the acoustic form of snake alarm calls is innate or learned remains unknown. It is possible that the unlearned/innate component played a substantial role in shaping such a relatively simple call that profoundly impacts fitness. The putative innate background of the call's acoustic features may have provided an opportunity for natural selection to mold the call properties. Future studies across multiples Paridae species are needed to address these hypotheses.

In summary, our study revealed that both the Varied tit and the Oriental tit exclusively used species-specific snake alarm calls in response to snakes, and their nestlings exhibited fledging responses to both heterospecific and conspecific snake alarm calls. We propose that these heterospecific nestling responses can be attributed to the overlap in certain acoustic properties of the two species' snake alarm calls, possibly tuned to the general acoustic sensitivity of nestlings' unlearned sensory templates specific to the family Paridae. Our hypothesis suggests that these sensitivities may have played a role in shaping the snake alarm call's acoustic properties responsible for eliciting nestling fledging responses. Notably, the acoustic overlap in snake calls between these two sympatric species, who otherwise differ in alarm calls to other predators, hints at the possibility that other species within the Paridae family may also possess snake-specific alarm calls with conserved acoustic similarities throughout their evolutionary history. Additionally, it is likely that their nestlings might respond similarly to both conspecific and heterospecific alarm calls. Therefore, we propose that future research should expand this experimental approach to multiple species across the Paridae phylogeny to uncover the specific shared acoustic properties of "snake calls”. Furthermore, investigating the detailed characteristics of putative templates in nestlings, not only in existing species but also in hypothetical ancestral species, can provide insights into the conservation of these properties from deep phylogenetic roots and their changes across various branches of the complete Paridae phylogeny. Beyond the Paridae family, a similar approach can be applied in the future to non-parid species with close phylogenetic relationships, aiming to determine if the putative similarities in key acoustic properties and nestling templates extend beyond the family boundaries.

## Methods

### Study site and subjects

The research was conducted on the Oriental tit and the Varied tit breeding in nest boxes (different number each year) in mixed forests of Seoul (three locations: 37°26′50′′N 126°56′44′′E, 37°35′46.1′′N 127°02′42.9′′E, 37°35′10.5′′N 127°06′03.9′′E), Republic of Korea. Based on monitoring of 100–200 nest boxes each year since 2011, we established that snakes are the main nest-predators for both species^25^. The field experiments were performed from 2013 to 2020 and comprised predator presentations and playback experiments.

### Predator presentation experiments

#### General information

From 2013 to 2020, we conducted predator presentation experiments on 23 breeding pairs of the Varied tit and 35 breeding pairs of the Oriental tit. In each experiment we recorded alarm calls of the pair (without differentiating between male and female due to practical reasons) in response to a presentation of a predator model (jay, cat, and snake). Next, we compared the alarm calls toward snake models with the alarm calls toward non-snake predator groups in order to determine if the snake-induced vocalizations are clearly different from the others.

#### Experimental design

In preliminary trials, we presented an empty cage on top of the nest box, and it did not induce any alarm calls: after a short period of initial cautiousness, the parents resumed their regular visits. We used four types of predator models to imitate the presence of a predator at a nest box: a live Steppe rat snake (*Elaphe dione*), a flexible plastic snake that resembled a live snake, a furry toy cat that resembled a domestic cat, and a stuffed Eurasian jay (*Garrulus glandarius*). The toy cat and the live and model snakes were placed on top of the nest box, while the stuffed jay was placed just next (within 20 cm) to the nest box (Table 1). All these predators (snake, jay, and cat) occur in the study area and are able to depredate nestlings and/or fledglings of small passerines. By using an attached transparent string to a body part of the predator model (neck of the plastic snake, tail of the furry toy cat, and leg of the Eurasian jay) we were able (hidden behind a tree or bush at a distance of 10–20 m) to imitate small movements of the snake body (rising up and lowering down frontal part of the body), cat’s tail (imitating a sitting cat that moves its tail) and jay’s head and upper body (imitating a bird looking around). Each breeding pair was tested with only one of the three predator treatments for the Varied tit (11 pairs in the plastic snake treatment, 5 pairs in the jay treatment, and 7 pairs in the cat treatment) and one of the four predator treatments for the Oriental tit (9 pairs in the live snake treatment, 10 pairs in the plastic snake treatment, 8 pairs in the jay treatment, and 8 pairs in the cat treatment). We used only one model per predator type, but the presentations varied randomly due to variability in the movements of a live snake and the movements of predator models induced manually. We aimed to test as many nests of each species as possible in each season while balancing the sample size and the distribution of testing dates among the treatment groups. Varying field conditions from year to year, such as the number of nests available and the rate of predation, ultimately determined the final sample sizes. For the same reason, we could not plan to randomly distribute the nests into treatment groups ahead of time, as it was practically impossible to know which nest will be available at the end of the breeding cycle. Finally, as the experiments were conducted in multiple years, we cannot formally exclude the possibility of pseudoreplication by conducting experiments on the same individuals in different years. However, considering a small sample size of experimental pairs among the possible number of pairs in the study area (roughly 50 pairs per year; Ha et al., unpublished data), we believe that the experiments can yield a valid dataset pertaining to the original purpose of the study.

#### Recording equipment and recording conditions

We used two different microphones (Primo EM172 and Sennheiser me67) which were connected to one of the three audio recorders (Tascam DR-05, Tascam DR-100, and Marantz pmd661 mk2) (see Table 1). We mounted the recording equipment on a 0.5 m high tripod and placed it 1 m away from the nest box. After the setup, the researcher waited 30 m away from the nest box until parent(s) entered the nest to became assured that the birds were habituated to the presence of the recording equipment. When the parents left their nest, we approached the nest box to set up a predator model on top of the box or within 20 cm from the box on one side of the nest box (jay). At their return, the alarm calls of the parents were recorded for 4 min from their first vocal response at 48 kHz sampling rate and 24-bit depth for jay presentations to the Oriental tit, and 96 kHz sampling rate and 24-bit depth for all the other presentations. At each presentation, we moved the predator model from a distance by using the string attached to the model. The movements of the model were repeated by pulling the string twice within a 10-s interval, followed by a release after an additional 5-s pause (for a visual demonstration, please refer to Supplementary Video V4). Based on our field experiences, we believe that these repeatable movements of the predator model are essential to induce clear alarming response of birds similar to natural responses to real predators. It was impossible to perform the experiments blind with respect to the predator type, study species, or nest ID. However, fixed procedures were implemented for moving the predator models by the experimenter, and standard methods were used for recording the bird responses, in order to avoid biases in the results. The presentations were conducted between 9:00 am and 5:00 pm, and rainy days were excluded from the experimental schedule.

### Playback experiments

#### General information

We used two playback treatments representing snake alarm calls (snake-induced alarm calls) of the Oriental tit and snake alarm calls of the Varied tit. We played the calls at the nests of the two species at the time when nestlings are sufficiently old to be able to jump out of the nest. This allowed us to test if nestlings of each species fledge upon hearing the conspecific as well as heterospecific snake alarm calls. The experiments were performed from 2018 to 2020.

#### Experimental design

When the nestlings in a nest reached the age at which fledging can be induced (more than 14 days old), we played back the recordings of conspecific or heterospecific alarm calls to them. Thus, only nests with nestlings that survived until 15 days after hatching were included in the playback experiment. Our previous research on the Oriental tit in this study area has shown that nestlings older than 14 days old are capable of fledging when they hear conspecific "snake call"^17^. We carried out playback tests on 30 nests of the Oriental tit and 20 nests of the Varied tit; 15 Oriental tit broods and 10 Varied tit broods were tested with the Oriental tit playback, and the same number of broods for each species were tested with the Varied tit playback. We used 5 different playback stimuli for each species. Hence, each playback stimulus was used at 3 broods of the Oriental tit and at 2 broods of the Varied tit. Even though multiple uses of the same playback cannot provide full independence between tests, we followed this method to assure that we use only the best quality recordings without excessive noises or alarm calls of the other individuals, or species, except the parents of the nest. We believed that using clear signals (with relatively low noise level) to induce nestlings’ response expected in a natural situation of vocalizing parents is more important than keeping formal independence of all data. This design cannot be formally used to statistically generalize the results to the responses of nestlings to a whole population of alarm calls of each species in the study area. Despite this drawback, the main difference between the two sets of these good-quality recordings is the species from which they were recorded (the Oriental tit or the Varied tit). Therefore, we think that we can use the results as indicators to assess if the alarm calls from the two species trigger similar or different responses in nestlings of the two species. For each species, we applied the 'minimization' randomization approach, as outlined by the guidance for Animal Research: Reporting of In Vivo Experiments (ARRIVE)^42^. In this method, the subsequent allocation of playback stimuli to nests was influenced by prior allocations, with the objective of mitigating potential biases associated with testing dates and sites in the field experiment. Specifically, we employed a rotation of treatments to ensure a balanced distribution of sample sizes across various dates to achieve robustness in the study's outcomes.

#### The playback stimuli

The playback samples were created from the recordings of predator presentations in the current study (see section "predator presentation experiment" above). The preparation of acoustic stimuli generally followed the protocol used in the previous study^17^. We created 3-min long playback stimuli by using alarm call recordings from plastic snake presentations for the Varied tit and from live snake presentations for the Oriental tit (see Supplementary Audios S1, S2). Our preliminary observations of the Varied tit confirmed that parental vocalizations in response to the plastic snake trigger fledging of the Varied tit nestlings, the same as Oriental tit vocalizations to live and artificial snakes trigger fledging of Oriental tit nestlings. We selected the five best recordings (i.e. those with the highest signal-to-noise ratio) for each species. From the 4 min-long recordings, we removed sections with following issues; excessive background noise, vocalizations of other species, overlapped calls by multiple individuals. We then removed sections without any vocalizations and fixed the maximum inter-phrase duration to one second to control the intensity of the alarm calls in the playback stimuli. This filtering procedure produced an audio file where inter-phrase durations do not exceed one second, and that only includes alarm call phrases from the parents of a specific nest. From this file, we chose the first minute and applied a 1 kHz high-pass filter. Finally, to create a 3-min playback stimulus, we converted the 1-min recording to 48 kHz sampling rate, 24-bit depth, and repeated it three consecutive times in the playback stimulus (Supplementary Fig. S4). We produced 5 playback stimuli from each species in this way. Our method allowed the playback stimuli to retain within and between species variation in acoustic properties of phrases among the recordings, while maintaining similar temporary dynamics of phrase delivery among the playback stimuli. Based on previous studies that revealed only alarm calls induced by snakes can trigger the fledging response, we did not include control playback groups in the experimental design. This allowed us to minimize the total number of nests required for the study.

#### Field procedures

We followed the protocol used in our previous study^17^, in which we proved that the jumping-out reactions of nestlings do not happen at all in response to ‘churr’-based alarm vocalizations of the Oriental tit to a mammal brood predator – chipmunk, while it does happen in response to snake alarm calls. This protocol considers that in the field situation there is no way of knowing for each nest at what age exactly the nestlings are old enough to be capable of jumping out of the nest box. Depending on the rate of growth and maturation of the brood, it may happen on day 15, 17, 18, or maybe even later. The protocol described below assures that the “no jumping out” outcome of the test procedure is not due to the nestlings being too young, but at the same time the protocol minimizes the chances that the nestlings fledge naturally before the researcher arrives at the nest to conduct the playback test. This protocol has proven effective in properly differentiating between calls that trigger nestlings to jump out of the nest and those that do not^17^.

Playback trials were performed between 9:00 am and 5:00 pm during non-rainy days, and the stimuli were played through a portable speaker (JBLAB HRS-20XB) when the parents were away from the nest. The speaker was positioned at the sidewall of a nest box. The researcher was standing 10-15 m away behind a bush or tree hiding from the view of the parents or from nestlings potentially jumping out of the box. Nestlings’ response to the playback was recorded as a binary variable (i.e. fledging response present or absent). We stopped a playback trial when the nestlings started to fledge and to minimize negative effects on nestlings we captured and put back the nestlings to the nest if they were too young to be able to properly fly. If parents arrived during the presentation of the playback, we stopped the playback and resumed it about five minutes after the parents left the area. We then repeated the same trial from the beginning of the playback until we successfully finished playing the 3-min playback stimulus without interruption by parental visits, or until we observed the first behavioral signs of fledging by nestlings (a nestling sitting in the nest box entrance). When this procedure did not result in fledging of the nestlings on one day, then we repeated the playback procedure on the next day in the same manner, and we repeated it again and again on the following days if no fledging was observed on a preceding day. This procedure was applied from the time when nestlings are 15 days old until the day when nestlings fledged in response to playback or until we found an empty nest indicating the likely natural fledging event.

A brood that showed signs of fledging during this procedure was assigned a binary value “response present”, and a brood that showed no signs of fledging until the nest became empty (presumably due to natural fledging) was assigned the value “response absent”. In the playback experiment, we defined the experimental units as the nests of the target species. The experiments could not have been conducted blind with respect to the experimental treatment or the nest ID/nest location. However, the potential for bias in the recorded variable values remains minimal due to the unequivocal binary nature of the response variable—yielding a 'yes/no' outcome, which has no chance of being subject to interpretation errors. Additionally, as described in “*Experimental design*”, an effort was made to ensure balanced sample size within each individual species, considering the distribution of testing dates and sites throughout the playback treatments.

### Sound analysis and statistical analysis

#### Syllable types and their frequency in vocal responses

We aimed to determine whether vocal responses of the Varied tit and the Oriental tit to snakes differed from their vocal responses to other predators. First, for each species, we visually inspected a 2-min sonogram recording from the initial call to classify syllables into distinct types based on their unique appearance. We then assigned a different letter from the English alphabet to each syllable type (Fig. 1; Supplementary Table S5 contains our syllable type symbols matched with the syllable type names previously used in the literature). For each syllable type we summed the total duration of all syllables in the initial 2 min of response in each recording. Then, we calculated the proportion of time that each syllable type occupied in the total duration of the vocalization per recording (duration for all syllable types). Analyzing these proportions allowed us to identify syllable types (for each species separately) that were predominantly used in responses to specific predators, including those syllables that comprised majority of time in vocalizations triggered by a snake, thus constituting what we refer to as ‘snake alarm calls’. Our classification of snake alarm call syllables closely matched the sonograms of snake alarm calls described in previous studies^15,17^. We decided to base the proportions on time (duration) rather than number of syllables to more precisely determine the syllable types that stimulate the receivers for most of the alarm vocalization duration.

#### Statistical comparisons of acoustic properties of syllable types present in snake alarm calls

Oriental tits used the broadly defined ‘LF/churr/rattle/jar’ type of syllable^17,19,20^ (labeled as type ‘L’ in this study) in alarm calls to all three predators. In snake presentations this was the only type of syllable used by the Oriental tit parents. Visual inspection of sonograms combined with auditory inspection of recordings is often sufficient to further categorize this type as either the typical ‘jar’ call characteristic for snake alarm calls^15,43^ or the other ‘churr’ sub-type used toward other predators (e.g. chipmunks)^17^. However, in some cases the difference is more subtle, especially based only on visual inspection of sonograms. Therefore, we statistically compared acoustic properties of this broad syllable type (i.e. syllable type ‘L’) between different predator presentations.

We also compared the acoustic properties of snake alarm call phrases (syllable type ‘L’ in the Oriental tit and syllable type ‘f’ in the Varied tit) between species. Here, the unit of analysis was a phrase composed of only the syllable type used in responses to snakes: the ‘L’ syllable type (for the Oriental tit) or only the ‘f’ syllable type (for the Varied tit). For these comparisons, we measured 13 acoustic properties of each phrase in the initial 2 min of each recording, including: Number of Syllables, Phrase Duration, Average Syllable Duration, Standard Deviation of Syllable Duration, Average Calling Rate, Standard Deviation of Calling Rate, Average Inter-syllable Interval, Average Center Frequency of Syllables within a Phrase, Standard Deviation of Center Frequency of Syllables within a Phrase, Average 1st Quartile of Frequency of Syllables within a Phrase, Average 3rd Quartile of Frequency of Syllables within a Phrase, Average Inter-quartile Frequency, Standard Deviation of Inter-quartile Range Frequency of Syllables within a Phrase. We then grouped these variables into 7 “duration variables” (or “timing variables”) and 6 “frequency variables” (Supplementary Table S6). These variables represent the “outcome measures” in the ARRIVE guidelines^42^. The measurements of acoustic variables were performed in Raven Pro 1.6^44^ with spectrogram configuration set at a Hann sample window size of 512, 256-sample hop size with 50% overlap, and frequency grid spacing and DFT size were set to 86.1 Hz and 512 samples. As these variables are often correlated with each other, we used parallel analysis (function ‘fa.parallel’)^45^ and principal components analysis (function ‘principal’)^45^ with varimax rotation to extract composite variables (principal components). Next, we conducted statistical analysis (Generalized Additive Model for Location, Scale and Shape, function 'gamlss')^46^ to test if the acoustic properties (the principal components) of the specific types of syllables (syllable type ‘L’ of the Oriental tit and syllable type ‘f’ of the Varied tit) are different among predators or between the two species. The goodness of fit of the models was assessed using a worm plot (function 'wp' in R package 'gamlss')^46^, which generated residual quantiles that aligned with a uniform distribution, indicating a reasonable fit of the model to the data. The analysis of ‘L’ syllables of the Oriental tit was based on n = 322 phrases from 10 nests in response to a plastic snake, n = 229 phrases from 8 nests in response to a jay, and n = 196 phrases from 8 nests in response to a cat. The analysis of ‘f’ syllables of the Varied tit was based on n = 288 phrases from 11 nests in response to a plastic snake. Additionally, we conducted variance component analysis (function 'anovaVCA' in R package 'VCA')^47^ for each principal component to explore the variability of acoustical properties by levels of explanatory or random variables (e.g., predator, species, and nest). All the statistical analyses were conducted in R^48^ (see Supplementary Table S7 for the basic information of acoustical dataset).

As we used two types of microphones that may differ in their frequency response curves, comparisons involving frequency variables can only be conducted within the set of data obtained using the same microphone model. Therefore, we decided to conduct all statistical comparisons within the same microphone model. Hence, we conducted three separate statistical analyses (Table 4): comparison of the Oriental tit’s ‘L’ syllables in snake calls with those in jay alarm calls (Sennheiser me67 microphone) and with those in cat alarm calls (Primo EM172 microphone), as well as comparison of snake alarm call syllables (‘L’ for Oriental tits and ‘f’ for Varied tits) between species (Primo EM172 microphone).

**Table 4:** Summary of the experimental schemes of predator presentation experiments and the equipment used in the recording of the vocalizations**.** The microphone type rather than the audio recorder model may affect the representation of frequencies in the recording. Therefore, we marked the microphone type in bold here to indicate the differences in recording equipment. This is also associated with either using a natural snake or plastic snake in the tests; as the natural snake movements during a test were less consistent across the tests than the imitated movements of the plastic snake we expect that variation among tests might have been larger for tests with natural snakes (hence, in experiments recorded using Sennheiser ME67). This determined the method of statistical comparisons of acoustic properties of ‘L’ phrases in the Oriental tit. Only the comparisons within the same microphone type were conducted. More details are in Supplementary Table S7.

Subject species	Recording equipment (**microphone,** audio recorder)	Predator model	Sample size
Oriental tit	**Primo EM172** Tascam DR-05	Plastic snake	10
		Furry toy cat	8
	**Sennheiser me67** Tascam DR-100	Stuffed Eurasian jay	8
	**Sennheiser me67** Marantz pmd661 mk2	Live snake	9
Varied tit	**Primo EM172** Tascam DR-05	Plastic snake	11
		Furry toy cat	7
		Stuffed Eurasian jay	5

#### Statistical analysis of playback experiments

When analyzing the result of playback experiments, we used Fisher's exact test (function ‘fisher test from the stats package in R’)^48^ and Mixed effects logistic regression (function ‘gamlss’)^46^ to compare the fledging responses between nestlings of two different species induced by playbacks of conspecific and heterospecifics snake alarm calls. In the logistic regression, we used the fledging response of nestlings (binary: 0 & 1) as the dependent variable and the nestling species (categorical with two levels: Varied tit nestlings & Oriental tit nestlings) as the explanatory variable. All the statistical analyses were conducted in R^48^.

### Approval for animal experiments

The experimental procedures were approved by the Institutional Animal Care and Use Committee of Seoul National University: SNU-180205-1, SNU-190413-2, and SNU-200413-1-1. All methods were carried out in accordance with relevant guidelines and regulations. We also confirm that we followed the ARRIVE guidelines.

### Supplementary Information

Supplementary Information 1.

Supplementary Video 1.

Supplementary Video 2.

Supplementary Video 3.

Supplementary Video 4.

Supplementary Information 2.

Supplementary Information 3.

## Data Availability

The datasets generated and analyzed during the current study are available from the authors (specifically the first author, JH, at wjdans1021@gmail.com) upon request.

## References

[1] Haff TM, Magrath RD (2012). Learning to listen? Nestling response to heterospecific alarm calls. Anim. Behav..

[2] Magrath RD, Haff TM, Fallow PM, Radford AN (2015). Eavesdropping on heterospecific alarm calls: From mechanisms to consequences. Biol. Rev..

[3] Potvin DA, Ratnayake CP, Radford AN, Magrath RD (2018). Birds learn socially to recognize heterospecific alarm calls by acoustic association. Curr. Biol..

[4] Magrath RD, Pitcher BJ, Gardner JL (2009). Recognition of other species' aerial alarm calls: Speaking the same language or learning another?. Proc. R. Soc. B-Biol. Sci..

[5] Magrath RD, Bennett TH (2012). A micro-geography of fear: Learning to eavesdrop on alarm calls of neighbouring heterospecifics. Proc. R. Soc. B-Biol. Sci..

[6] Wheatcroft D, Price TD (2013). Learning and signal copying facilitate communication among bird species. Proc. R. Soc. B-Biol. Sci..

[7] Johnson FR, McNaughton EJ, Shelley CD, Blumstein DT (2003). Mechanisms of heterospecific recognition in avian mobbing calls. Aust. J. Zool..

[8] Davies NB, Madden JR, Butchart SHM, Rutila J (2006). A host-race of the cuckoo *Cuculus*
*canorus* with nestlings attuned to the parental alarm calls of the host species. Proc. R. Soc. B-Biol. Sci..

[9] Nieder A, Mooney R (2020). The neurobiology of innate, volitional and learned vocalizations in mammals and birds. Philos. Trans. R. Soc. B-Biol. Sci..

[10] Wheeler BC, Fahy M, Tiddi B (2019). Experimental evidence for heterospecific alarm signal recognition via associative learning in wild capuchin monkeys. Anim. Cogn..

[11] Keen SC, Cole EF, Sheehan MJ, Sheldon BC (2020). Social learning of acoustic anti-predator cues occurs between wild bird species. Proc. R. Soc. B-Biol. Sci..

[12] Langham GM, Contreras TA, Sieving KE (2006). Why pishing works: Titmouse (Paridae) scolds elicit a generalized response in bird communities. Ecoscience.

[13] Fallow PM, Gardner JL, Magrath RD (2011). Sound familiar? Acoustic similarity provokes responses to unfamiliar heterospecific alarm calls. Behav. Ecol..

[14] Randler C (2012). A possible phylogenetically conserved urgency response of great tits (*Parus** major*) towards allopatric mobbing calls. Behav. Ecol. Sociobiol..

[15] Suzuki TN (2011). Parental alarm calls warn nestlings about different predatory threats. Curr. Biol..

[16] Suzuki TN (2015). Assessment of predation risk through referential communication in incubating birds. Sci. Rep..

[17] Ha J (2020). Experimental study of alarm calls of the oriental tit (*Parus** minor*) toward different predators and reactions they induce in nestlings. Ethology.

[18] Billerman, S. M., Keeney, B. K., Rodewald, P. G. & Schulenberg, T. S. *Birds of the world*. (Cornell Laboratory of Ornithology, 2020).

[19] Hailman JP (1989). The organization of major vocalizations in the Paridae. Wilson Bull..

[20] Carlson NV, Healy SD, Templeton CN (2017). A comparative study of how British tits encode predator threat in their mobbing calls. Anim. Behav..

[21] Yu J, Xing X, Jiang Y, Liang W, Wang H, Møller AP (2017). Alarm call-based discrimination between common cuckoo and Eurasian sparrowhawk in a Chinese population of great tits. Ethology.

[22] Zhang L, Yu J, Shen C, Yin D, Jin L, Liang W, Wang H (2022). Geographic variation in note types of alarm calls in Japanese tits (*Parus** minor*). Animals.

[23] Gill SA, Bierema AMK (2013). On the meaning of alarm calls: A review of functional reference in avian alarm calling. Ethology.

[24] Johansson US, Nylinder S, Ohlson JI, Tietze DT (2018). Reconstruction of the late Miocene biogeographical history of tits and chickadees (Aves: Passeriformes: Paridae): A comparison between discrete area analyses and probabilistic diffusion approach. J. Biogeog..

[25] Ha J (2022). Nest predation and alarm call communication in two bird species of the Paridae.

[26] Suzuki TN (2014). Communication about predator type by a bird using discrete, graded and combinatorial variation in alarm calls. Anim. Behav..

[27] Bartmess-LeVasseur J, Branch CL, Browning SA, Owens JL, Freeberg TM (2010). Predator stimuli and calling behavior of Carolina chickadees (*Poecile*
*carolinensis*), Tufted titmice (*Baeolophus*
*bicolor*), and White-breasted nuthatches (*Sitta*
*carolinensis*). Behav. Ecol. Sociobiol..

[28] Courter JR, Ritchison G (2010). Alarm calls of tufted titmice convey information about predator size and threat. Behav. Ecol..

[29] Fallow PM, Pitcher BJ, Magrath RD (2013). Alarming features: Birds use specific acoustic properties to identify heterospecific alarm calls. Proc. R. Soc. B-Biol. Sci..

[30] Ghirlanda S, Enquist M (2003). A century of generalization. Anim. Behav..

[31] Searcy WA, Nowicki S (2003). The evolution of animal communication: reliability and deception in signaling systems.

[32] Kagel JH, Battalio RC, Green L (1995). Economic choice theory: An experimental analysis of animal behavior.

[33] Blumstein DT, Munos O (2005). Individual, age and sex-specific information is contained in Yellow-bellied marmot alarm calls. Anim. Behav..

[34] Leavesley AJ, Magrath RD (2005). Communicating about danger: urgency alarm calling in a bird. Anim. Behav..

[35] Wiley RH (1982). Adaptations for acoustic communication in birds: sound transmission and signal detection. Acoust. Comm. Birds.

[36] Ten Cate C, Rowe C (2007). Biases in signal evolution: Learning makes a difference. Trends Ecol. Evol..

[37] Fallow PM, Magrath RD (2010). Eavesdropping on other species: Mutual interspecific understanding of urgency information in avian alarm calls. Anim. Behav..

[38] Manser MB (2001). The acoustic structure of suricates' alarm calls varies with predator type and the level of response urgency. Proc. R. Soc. B-Biol. Sci..

[39] Zuberbühler K (2001). Predator-specific alarm calls in Campbell's monkeys, Cercopithecus campbelli. Behav. Ecol. Sociobiol..

[40] Fasanella M, Fernández GJ (2009). Alarm calls of the Southern House Wren *Troglodytes **musculus*: Variation with nesting stage and predator model. J. Ornithol..

[41] Yorzinski JL, Vehrencamp SL (2009). The effect of predator type and danger level on the mob calls of the American crow. Condor.

[42] Percie du Sert N (2020). The ARRIVE guidelines 2.0: updated guidelines for reporting animal research. PLoS Biol..

[43] Ha J (2018). Effect of nestlings’ age on parental responses to a predatory snake in Parus minor. Behaviour.

[44] K. Lisa Yang Center for Conservation Bioacoustics. Raven Pro: Interactive Sound Analysis Software (Version 1.6). https://ravensoundsoftware.com/ (2021).

[45] Revelle, W. psych: Procedures for Personality and Psychological Research. https://CRAN.R-project.org/package=psych/ (2021).

[46] Rigby RA, Stasinopoulos DM (2005). Generalized additive models for location, scale and shape. J. R. Stat. Soc. Ser. C-Appl. Stat..

[47] Andre, S. & Florian, D. VCA: Variance Component Analysis. R package version 1.4.3. https://CRAN.R-project.org/package=VCA/ (2020).

[48] R Core Team. R: A language and environment for statistical computing. https://www.R-project.org/ (2020).

