# Supplementary material for: Unlearned adaptive responses to heterospecific referential alarm calls in two bird species from separate evolutionary lineages
Source: Sci Rep. 2023 Nov 20;13:20287. doi: 10.1038/s41598-023-47052-5 (PMC10662011; doi:10.1038/s41598-023-47052-5)
Supplement: Supplementary file 1 — Supplementary Information 1. [file 41598_2023_47052_MOESM1_ESM.pdf]

SUPPLEMENTARY MATERIALS TO:

Unlearned adaptive responses to heterospecific referential alarm calls in two bird species from separate evolutionary lineages

Jungmoon Ha[1], Keesan Lee[1], Eunjeong Yang[1], Woojoo Kim[1], Hokyung Song[2], Injae Hwang[3], Larisa Lee-Cruz[4], Jinseok Park[1], Jihyeon Song[1], Chan-ryul Park[5], Wooshin Lee[6], Piotr Jablonski[1,7]*, Sang-im Lee[8]*

* Corresponding authors

Affiliations:

1. Laboratory of Behavioral Ecology and Evolution, School of Biological Science, Seoul National University, Seoul, Republic of Korea
2. Jeju National University, Jeju-si, Jeju-do, Republic of Korea
3. National Institute of Biological Resources, Seo-gu, Incheon, Republic of Korea
4. INRAE, UMR TETIS, Maison de la Télédétection, 500 Rue Jean François Breton, 34090 Montpellier, France
5. Urban Forests Division, National Institute of Forest Science (NIFoS), Dongdaemun-gu, Seoul, Republic of Korea
6. Department of Forest Sciences, CALS, Seoul National University, Seoul, Republic of Korea
7. Behavioral Ecology Group, Museum and Institute of Zoology, Polish Academy of Sciences, Warsaw, Poland
8. Laboratory of Integrative Animal Ecology, Department of New Biology, DGIST, Korea

CONTENT:

PART 1: SYLLABLE TYPES AND THEIR USE IN VOCALIZATIONS

Supplementary Figure S1. Example sonograms of 'L' syllable phrases to different predators for the Oriental tit and 'f' syllable phrases to snake presentations for the Varied tit. ········ 3

PART 2: ACOUSTIC ANALYSES OF SYLLABLE TYPE "churr" IN THE TARGET SPECIES AND RESULT DATASET OF PLAYBACK EXPERIMMENT

Supplementary Table S1. Summary of PCA (varimax-rotated principal components) and statistical analysis comparing 'L' syllables of the Oriental tit between the live snake and the jay presentations recorded with Sennheiser me67 microphone (Table 4 in Methods). ········ 4

Supplementary Table S2. Summary of varimax-rotated principal component analysis (PCA) and statistical analysis comparing 'L' syllables between the plastic snake and the toy cat presentations to Oriental tits recorded with Primo EM172 microphone (Table 4 in Methods). ········ 5

Supplementary Figure S2. Comparisons of acoustic variables describing the syllable type 'L' phrases of the Oriental tit between the different predator treatments recorded using two different microphones (which might have affected frequency variables) and compared withing pairs that come from recordings with the same type of microphone: jay vs. live snake (*Sennheiser*), plastic snake vs. furry toy cat (*Primo*). ········ 6

Supplementary Table S3. Summary of PCA (varimax-rotated principal components) and statistical model analysis comparing 'L' syllables of the Oriental tit and 'f' syllables of the Varied tit in response to the plastic snake (here we used the term 'LF/churr' syllable referring to both 'L' and 'f' syllables). ········ 7

Supplementary Figure S3. Boxplots for acoustic variables of 'L' phrases of the Oriental tit (OT) in response to the plastic snake and 'f' phrases of the Varied tit (VT) in response to the plastic snake. ········ 8

Supplementary Table S4. Results of playback experiments using two playback treatments toward nestlings of the Oriental tit and the Varied tit. 9

PART 3: LIST OF SUPPLEMENTARY MEDIA FILES WITH EXPLANATIONS

Supplementary Audio S1. One of the playback samples for the snake alarm calls of the Oriental tit. 10

Supplementary Audio S2. One of the playback samples for the snake alarm calls of the Varied tit. 10

Supplementary Video V1. Examples of alarm calls of the Oriental tit and the Varied tit toward a toy cat. 10

Supplementary Video V2. Examples of alarm calls of the Oriental tit and the Varied tit toward a stuffed jay. 10

Supplementary Video V3. Examples of alarm calls of the Oriental tit and the Varied tit toward a snake. 10

Supplementary Video V4. Examples of predator stimuli, including a stuffed Eurasian jay, a furry toy cat, and a plastic snake, demonstrating their movements as used in the predator presentation experiment. 10

PART 4: ADDITIONAL INFORMATIONS ABOUT METHODS AND ACOUSTICAL DATASET

Supplementary Figure S4. Examples of sonograms showing a schema of the preparation process of playback samples. .. 11

Supplementary Table S5. Symbols of syllable types of the Oriental tit in the current study matched with the syllable names previously used in the literature. 12

Supplementary Table S6. Abbreviations and definitions of acoustic variables calculated for each phrase in the vocal output recorded in response to the experimental presentations of predators. 13

Supplementary Table S7. Basic data structure for the analyses of detailed acoustic properties of phrases made of Oriental tits' ‘L’ and Varied tits' ‘f’ syllables: number of recordings (one recording per nest) and number of phrases recorded per recording in each of the different predator and species group. 14

SUPPLEMENTARY MATERIALS PART 1:
SYLLABLE TYPES AND THEIR USE IN VOCALIZATIONS

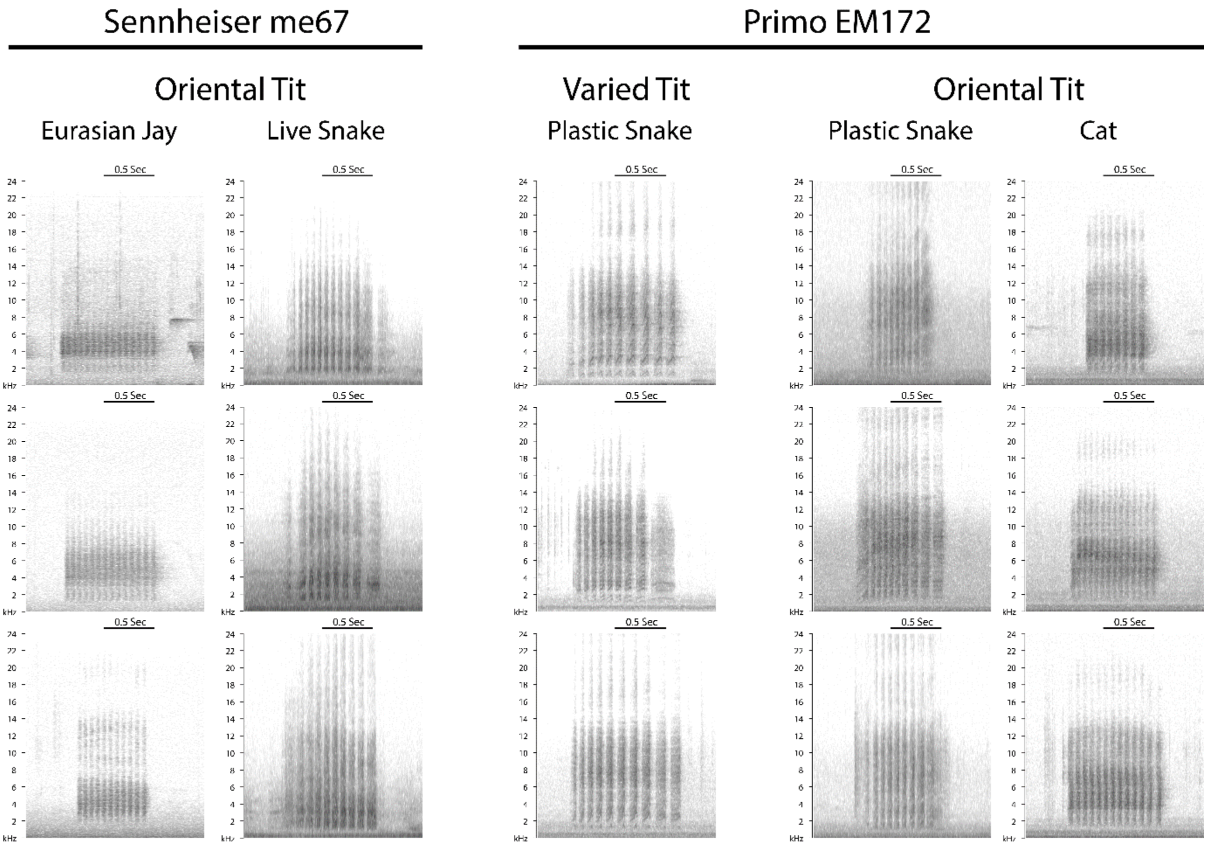

Supplementary Figure S1. Example sonograms of 'L' syllable phrases to different predators for the Oriental tit and 'f' syllable phrases to snake presentations for the Varied tit. Each sonogram was produced from a single phrase of a different pair. Note the visually perceptible higher similarity between snake alarm calls from the two species (i.e., between 'L' and 'f' syllables) and lower similarity between the 'L' syllables to snakes and the two other predators (Eurasian jay and cat).

SUPPLEMENTARY MATERIALS PART 2:
ACOUSTIC ANALYSES OF SYLLABLE TYPE 'L' ["churr" type] IN THE ORIENTAL TIT AND RESULT DATASET OF PLAYBACK EXPERIMMENT

Supplementary Table S1. Summary of PCA (varimax-rotated principal components) and statistical analysis comparing 'L' syllables of the Oriental tit between the live snake and the jay presentations recorded with Sennheiser me67 microphone (Table 4 in Methods). Three principal components were extracted from 13 variables (7 duration variables and 6 frequency variables; explanations of the acronyms are in Table S6). The columns of the principal components were arranged from largest to smallest proportion of variance explained by each component (% of variance explained shown in parentheses). Loadings higher than 0.5 are marked with grey background. The variables are grouped into "Duration variables" and "Frequency variables". The bottom of the table shows results of statistical comparison between snake and jay for each of the three PCs. This table corresponds to Fig. 2A & B and Table 1.

	**Standardized loadings in varimax-rotated principal components**		
**Acoustic variables**	**PC1: Frequency (32%)** *(pitch, range, variability)*	**PC2: Syllable (26%)** (*syllable duration+its variability & call rate+its variability*)	**PC3: Phrase (17%)** (*phrase duration, syllable number & call rate variability*)
**<u>Duration variables</u>**			
**NS**	0.13	-0.07	0.92
**PD**	0.17	0.39	0.81
**ASD**	0.05	0.91	0.03
**SDSD**	0.03	0.82	0.30
**ACR**	-0.18	-0.82	0.05
**SDCR**	0.05	0.59	0.58
**AISI**	-0.01	0.39	-0.39
**<u>Frequency variables</u>**			
**ACFS**	0.94	-0.17	-0.01
**SDCFS**	0.78	0.25	0.30
**A1QFS**	0.71	-0.42	-0.07
**A3QFS**	0.98	0.06	0.05
**AIRFS**	0.81	0.35	0.11
**SDIRFS**	0.64	0.33	0.20

**Component**	**Estimate (snake)**	**Standard error**	**p-value**	**Fitted distribution (link function)**
PC1: **Frequency**	5.425	0.031	<0.001	Power exponential (identity)
PC2: **Syllable**	4.207	0.087	<0.001	Johnson SU (identity)
PC3: **Phrase**	1.779	0.105	<0.001	Johnson SU (identity)

Biological meaning of the principal components:
PC1: This is an index of how high the frequency of a syllable is, how variable the frequency of syllables within a phrase is, and how wide the frequency range of a syllable is. Higher values of PC1 indicate higher frequency of 'L' syllables that vary more among the syllables within a phrase. It also indicates wider frequency range of a phrase composed of these syllables.
PC2: This is an index of how long and how variable is the duration of syllables in a phrase, and how fast syllables in a phrase were repeated. Higher values of PC2 indicate 'L' syllables of longer duration that varies more among syllables within a phrase. Higher PC2 also indicate 'L' syllables of slower repetition rate within a phrase that varies more among syllables within a phrase.
PC3: This is an index of how long duration of a phrase is, and how many syllables it contains. Higher values of PC3 indicate longer phrases of 'L' syllables that have more syllables in the phrase. Higher PC3 also indicate 'L' syllables of faster repetition within a phrase.

Supplementary Table S2. Summary of varimax-rotated principal component analysis (PCA) and statistical analysis comparing 'L' syllables between the plastic snake and the toy cat presentations to Oriental tits recorded with Primo EM172 microphone (Table 4 in Methods). Four principal components were extracted from 13 variables (7 duration variables and 6 frequency variables; explanations of the acronyms are in Table S6). The columns of the principal components were arranged from largest to smallest proportion of variance explained by each component (% of variance explained shown in parentheses). Loadings higher than 0.5 are marked with grey background. The variables are grouped into "Duration variables" and "Frequency variables". The bottom of the table shows results of statistical comparison between snake and cat for each of the three PCs. This table corresponds to Fig. 2C and 2D and Table 2.

	Standardized loadings in varimax-rotated principal components			
**Acoustic variable**	**PC1: Frequency (21%)** (*range & its variation*)	**PC2: Phrase (17%)** (*phrase duration, syllable number & call rate variation*)	**PC3: Syllable (24%)** (*syllable duration, variability, intervals & call rate*)	**PC4: Frequency: pitch (20%)** (*pitch*)
**Duration variable**				
**NS**	-0.02	0.91	-0.23	0.08
**PD**	0.12	0.91	0.29	0.13
**ASD**	0.15	0.05	0.92	0.05
**SDSD**	0.44	0.36	0.57	0.09
**ACR**	-0.09	0.09	-0.90	-0.21
**SDCR**	0.53	0.52	0.15	0.30
**AISI**	0.09	0.05	0.83	0.14
**Frequency variable**				
**ACFS**	0.38	0.09	0.21	0.88
**SDCFS**	0.84	0.14	0.01	-0.05
**A1QFS**	-0.06	0.23	0.06	0.93
**A3QFS**	0.54	0.01	0.32	0.76
**AIRFS**	0.77	-0.17	0.37	0.25
**SDIRFS**	0.72	0.10	0.12	0.39

**Component**	**Estimate (snake)**	**Standard error**	**p-value**	**Fitted distribution (link function)**
PC1: **Frequency**	6.155	0.114	<0.001	Johnson SU (identity)
PC2: **Phrase**	1.103	0.130	<0.001	Johnson SU (identity)
PC3: **Syllable**	4.094	0.084	<0.001	Johnson SU (identity)
PC4: **Frequency: pitch**	5.724	0.097	<0.001	Johnson SU (identity)

Biological meaning of the principal components:
PC1: This is an index of how wide and how variable is the inter-quartile frequency range of syllables within a phrase, and how variable is the center frequency of syllables within a phrase. Higher values of PC1 indicate 'L' syllables of higher inter-quartile frequency range that relatively varies more among syllables within a phrase. Higher PC1 values also indicates 'L' syllables of more variable center frequency within a phrase.
PC2: This is an index of how long is the duration of a phrase, and how many syllables there are in a phrase. Higher values of PC2 indicate longer phrases of 'L' syllables that have more syllables in the phrase.
PC3: This is an index of how long and how variable is the duration of syllables within a phrase, and how fast the syllables in a phrase were repeated. It is also an indicator of the inter-syllable duration. Higher values of PC3 indicate 'L' syllables of longer duration that relatively varies more among syllables within a phrase. Higher PC3 values also indicates 'L' syllables of slower repetition rate in a phrase that relatively has longer inter-syllable duration within a phrase.
PC4: This is an index of how high is the general frequency (pitch) of a syllable. Higher values of PC4 indicate 'L' syllables of higher center, first quartile, and third quartile frequency.

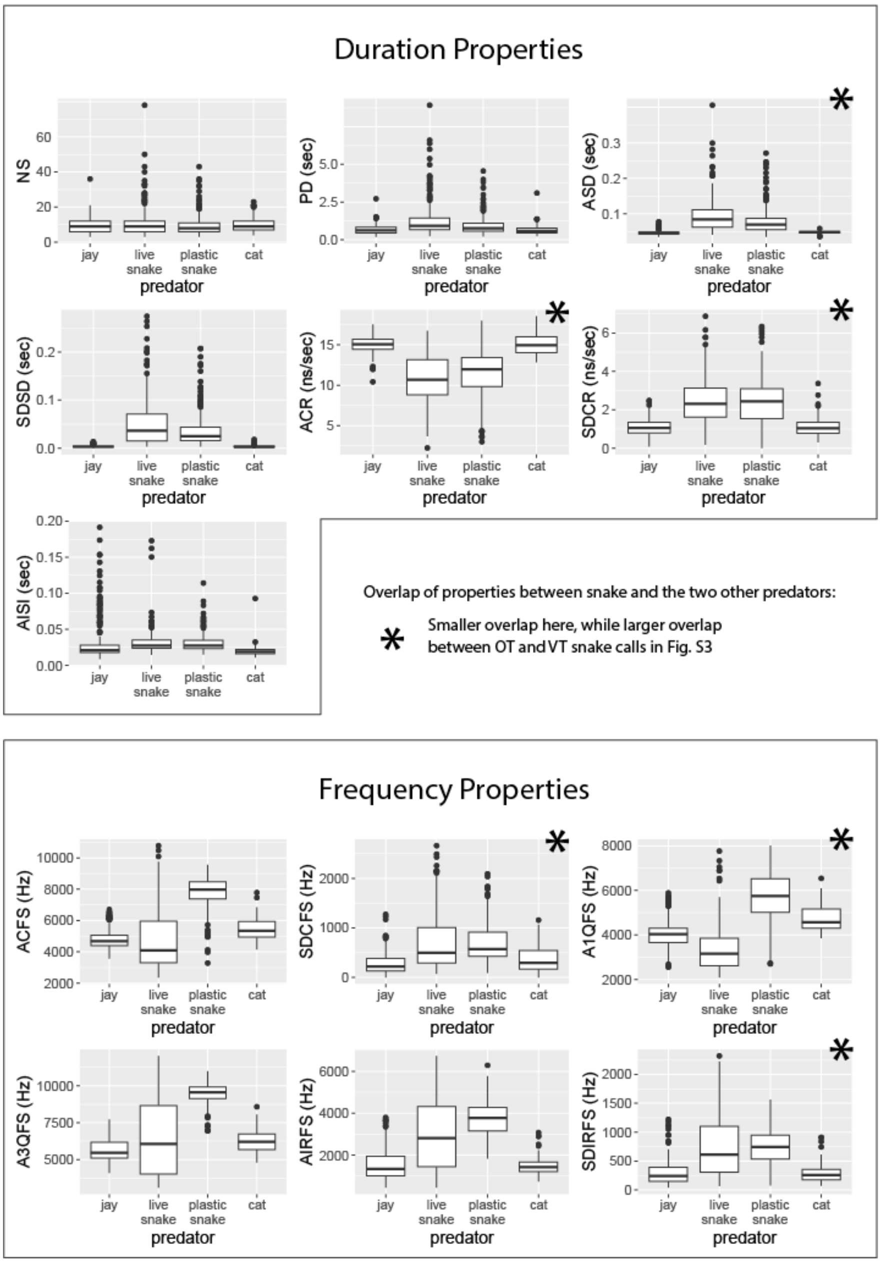

Supplementary Figure S2. Comparisons of acoustic variables describing the syllable type 'L' phrases of the Oriental tit between the different predator treatments recorded using two different microphones (which might have affected frequency variables) and compared withing pairs that come from recordings with the same type of microphone: jay vs. live snake (*Sennheiser*), plastic snake vs. furry toy cat (*Primo*). The two snake treatments (live snake and plastic snake) recorded with different microphones (see Table 4 in Methods) are next to each other in the center of a panel to visualize the differences between them: Sennheiser me 67 microphone was used for recordings of responses to jay and live snake (left side of each panel), and Primo EM172 microphone was used for recording responses to plastic snake and toy cat (right side of each panel). Filled asterisks indicate variables that show little overlap in jay-live snake and plastic snake-toy cat comparisons and extensive overlap in the Oriental Tit – Varied Tit snake alarm calls comparisons (Fig. S3). Please see Table S6 for full definitions, and calculation methods of the variables. Here are brief explanations:

<u>Duration variables:</u>

NS – **N**umber of **S**yllables; **PD** = **P**hrase **D**uration; **ASD*** – **A**verage **S**yllable **D**uration; **SDSD** – **S**tandard **D**eviation of **S**yllable **D**uration; **ACR*** – **A**verage **C**alling **R**ate; **SDCR*** – **S**tandard **D**eviation of **C**alling **R**ate; **AISI** - **A**verage **I**nter-**S**yllable **I**nterval.

<u>Frequency variables:</u>

ACFS – **A**verage **C**enter **F**requency of **S**yllables within a phrase; **SDCFS*** – **S**tandard **D**eviation of **C**enter **F**requency of **S**yllables within a phrase; A1QFS* – **A**verage **1**st **Q**uartile of **F**requency of **S**yllables within a phrase; **A3QFS** – **A**verage **3**rd **Q**uartile of **F**requency of **S**yllables within a phrase; **AIRFS** – **A**verage **I**nter-quartile **F**requency; **SDIEFS*** - **S**tandard **D**eviation of **I**nter-quartile **R**ange **F**requency of **S**yllables in a phrase.

Supplementary Table S3. Summary of PCA (varimax-rotated principal components) and statistical model analysis comparing 'L' syllables of the Oriental tit and 'f' syllables of the Varied tit in response to the plastic snake (here we used the term 'LF/churr' syllable referring to both 'L' and 'f' syllables). Four principal components were extracted from 13 variables (7 duration variables and 6 frequency variables; explanations of the acronyms are in Table S6). The columns of the principal components were arranged from largest to smallest proportion of variance explained by each component (% of variance explained shown in parentheses). Loadings higher than 0.5 are marked with grey background. The variables are grouped into "Duration variables" and "Frequency variables". The bottom of the table shows results of statistical comparison between the two species for each of the three PCs. This table corresponds to Fig. 3A, 3B, and Table 3.

	Standardized loadings in varimax-rotated principal components			
**Acoustic variable**	**PC1: Frequency: pitch (24%)** (*pitch*)	**PC2: Phrase (18%)** (*phrase duration, syllable number & call rate variability*)	**PC3: Syllable (17%)** (*syllable duration+its variability & call rate*)	**PC4: Frequency (14%)** (*range & pitch variation*)
**Duration variables**				
**NS**	-0.07	0.89	-0.12	-0.13
**PD**	-0.09	0.92	0.15	-0.10
**ASD**	0.15	-0.06	0.93	0.07
**SDSD**	0.31	0.27	0.59	0.38
**ACR**	0.11	0.16	-0.81	0.25
**SDCR**	0.31	0.56	-0.01	0.42
**AISI**	-0.42	0.09	0.53	-0.08
**Frequency variables**				
**ACFS**	0.97	0.00	0.00	0.03
**SDCFS**	-0.15	0.17	-0.08	0.79
**A1QFS**	0.91	0.16	0.01	-0.28
**A3QFS**	0.91	-0.10	0.03	0.24
**AIRFS**	0.10	-0.36	0.03	0.73
**SDIRFS**	0.09	0.39	-0.06	0.30
**Component**	**Estimate (VT)**	**Standard error**	**p-value**	**Fitted distribution (link function)**
PC1: **Frequency: pitch**	-3.895	0.096	<0.001	Johnson SU (identity)
PC2: **Phrase**	0.513	0.144	<0.001	Johnson SU (identity)
PC3: **Syllable**	0.334	0.089	<0.001	Johnson SU (identity)
PC4: **Frequency**	-1.776	0.108	<0.001	Johnson SU (identity)

Biological meaning of the principal components:
PC1: This is an index of how high is the general frequency (pitch) of a syllable. Higher values of PC1 indicate 'LF/churr' syllables of higher center, first quartile, and third quartile frequency.
PC2: This is an index of how long is the duration of a phrase, and how many syllables there are in a phrase. Higher values of PC2 indicate longer phrases that have more 'LF/churr' syllables. It also indicates higher variation of repetition rate of syllables within a phrase.
PC3: This is an index of how long and how variable is the duration of syllables within a phrase, and how fast the syllables in a phrase were repeated. Higher values of PC3 indicate 'LF/churr' syllables of longer duration that relatively varies more among syllables within a phrase. Higher values of PC3 also indicate 'LF/churr' syllables of slower repetition rate in a phrase with relatively longer inter-syllable duration.
PC4: This is an index of how wide is the inter-quartile frequency range of a syllable, and how variable is the center frequency of syllables in a phrase. Higher values of PC4 indicate 'LF/churr' syllables of wider inter-quartile frequency range and more variation of center frequency within a phrase.

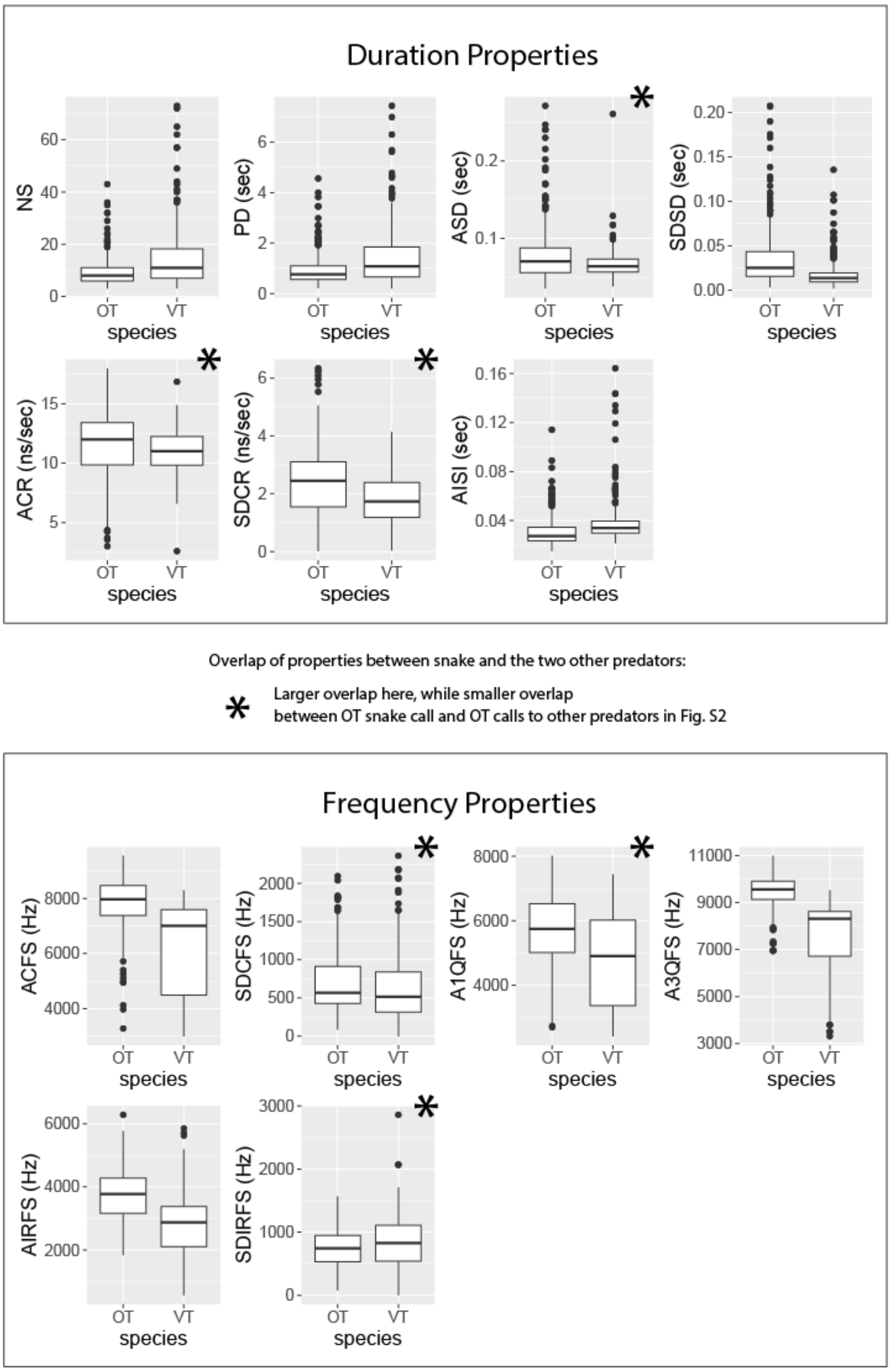

Supplementary Figure S3. Boxplots for acoustic variables of 'L' phrases of the Oriental tit (OT) in response to the plastic snake and 'f' phrases of the Varied tit (VT) in response to the plastic snake. Please see Table S6 for full definitions, and calculation methods of the variables. Here are brief explanations:

<u>Duration variables:</u>

NS – **N**umber of **S**yllables; **PD** = **P**hrase **D**uration; **ASD*** – **A**verage **S**yllable **D**uration; **SDSD** – **S**tandard **D**eviation of **S**yllable **D**uration; **ACR*** – **A**verage **C**alling **R**ate; **SDCR*** – **S**tandard **D**eviation of **C**alling **R**ate; **AISI** - **A**verage **I**nter-**S**yllable **I**nterval.

<u>Frequency variables:</u>

ACFS – **A**verage **C**enter **F**requency of **S**yllables within a phrase; **SDCFS*** – **S**tandard **D**eviation of **C**enter **F**requency of Syllables within a phrase; A1QFS* – **A**verage **1**st **Q**uartile of **F**requency of **S**yllables within a phrase; **A3QFS** – **A**verage **3**rd **Q**uartile of **F**requency of **S**yllables within a phrase; **AIRFS** – **A**verage **I**nter-quartile **F**requency; **SDIEFS*** - **S**tandard **D**eviation of **I**nter-quartile **R**ange **F**requency of **S**yllables in a phrase.

Supplementary Table S4. Results of playback experiments using two playback treatments toward nestlings of the Oriental tit and the Varied tit.

	**Nestling Species**					
	**Oriental tit**			**Varied tit**		
Playback Species	Fledged	Not Fledged	Total	Fledged	Not Fledged	Total
Oriental tit	13	2	15	10	0	10
Varied tit	10	5	15	10	0	10
Total	23	7	30	20	0	20

SUPPLEMENTARY MATERIALS PART 3:

LIST OF SUPPLEMENTARY MEDIA FILES WITH EXPLANATIONS

Supplementary Audio S1. One of the playback samples for the snake alarm calls of the Oriental tit.

Supplementary Audio S2. One of the playback samples for the snake alarm calls of the Varied tit.

Supplementary Video V1. Examples of alarm calls of the Oriental tit and the Varied tit toward a toy cat.

Supplementary Video V2. Examples of alarm calls of the Oriental tit and the Varied tit toward a stuffed jay.

Supplementary Video V3. Examples of alarm calls of the Oriental tit and the Varied tit toward a snake.

Supplementary Video V4. Examples of predator stimuli, including a stuffed Eurasian jay, a furry toy cat, and a plastic snake, demonstrating their movements as used in the predator presentation experiment.

SUPPLEMENTARY MATERIALS PART 4:
ADDITIONAL INFORMATIONS ABOUT METHODS

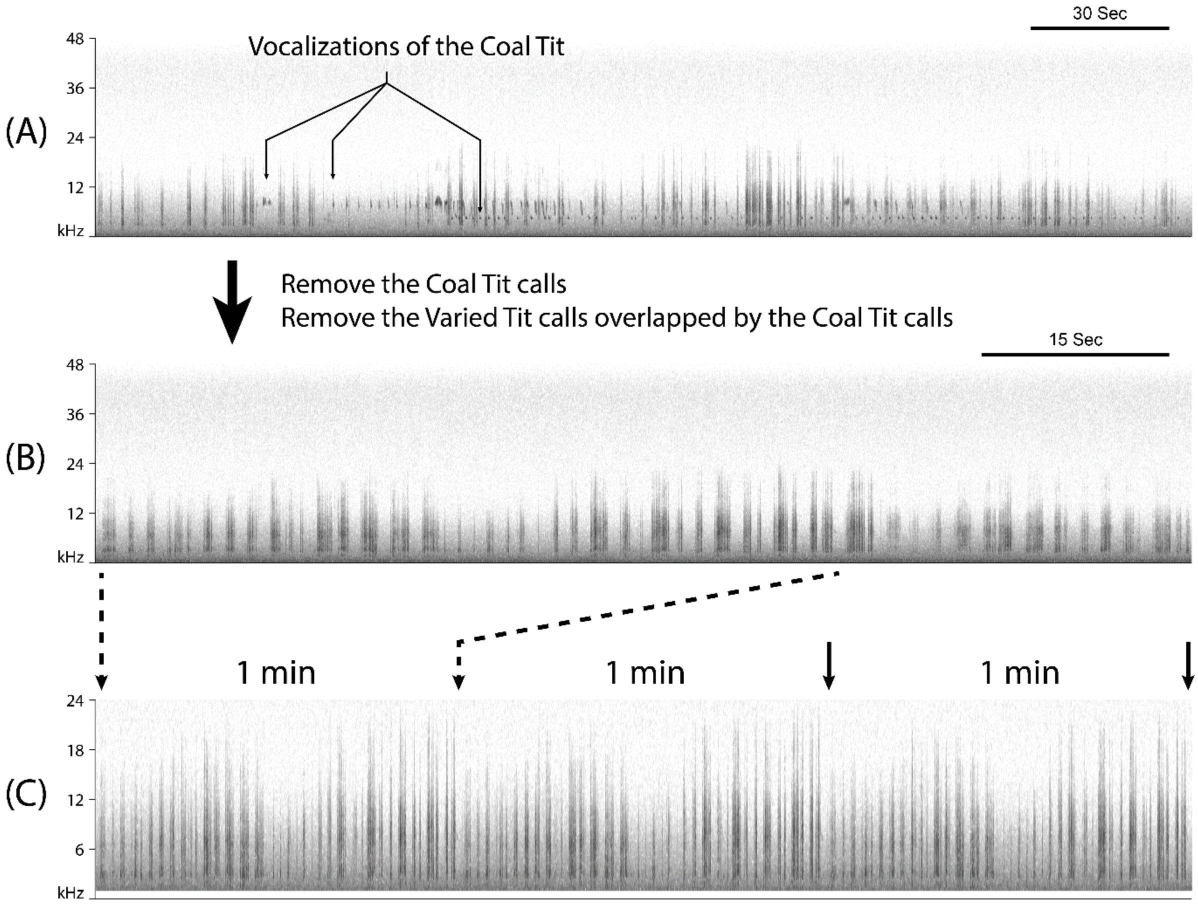

Supplementary Figure S4. Examples of sonograms showing a schema of the preparation process of playback samples. (A) The full 4-minutes recording of alarm calls from the Varied tit responded to the plastic snake model. One Coal tit was attracted to the alarm calls of the Varied tit and vocalized their own calls. (B) The same recording after deleting the alarm calls of the Coal tit and alarm calls of the Varied tit overlapped by the Coal tit calls. We also fixed the maximum inter-phrase duration to 1 second based on the timely characteristics of natural vocal responses in the recordings (interquartile range of inter-phrase duration is from 0.4 to 1.7 seconds) to control the intensity of the alarm calls at high level by removing sections with long silences. (C) The final 3-minutes playback sample. The first 1-minute of the (B) was filtered by a 1 kHz high-pass filter after it was converted to a 48 kHz sampling rate. Then the 1-minute section was repeated three times to produce the final 3-minutes playback sample.

Supplementary Table S5. Symbols of syllable types of the Oriental tit in the current study matched with the syllable names previously used in the literature. The table shows the names of syllable types for the Great tit and the Oriental tit previously used in the literature and the corresponding syllable types distinguished in our study (see sonograms of different syllable types in our study presented in the Figure 1).

Previous studies	Study species of the research	Expression of syllable types in the literature	Syllable type symbols of the Oriental tit in the current study
Hinde 1952	*Parus major*	'pit' 'dit' 'chat' 'twink'	'A' 'B' 'C' 'D' 'E' 'f' 'G'
		'dur'	'K'
		'churr' 'chich'	'L'
Hailman 1989	*Parus major*	'HF-notes'	'A' 'B' 'C' 'D' 'E' 'f' 'G'
		'IF-notes'	'H' 'I'
		'LF-notes'	'J' 'K'
		'LF-notes' 'churr'	'L'
Carlson et al. 2018	*Parus major*	'tonal'	'A' 'B' 'C' 'D' 'E' 'f' 'G'
		'chirp'	'H'
		'jar/rattle/D'	'L'
Suzuki 2011,2014,2015	*Parus minor*	'chicka'	'A' 'B' 'C' 'D' 'E' 'f' 'G'
		'jar'	'L'
Ha et al. 2018	*Parus minor*	'jar'	'L'
Ha et al. 2020	*Parus minor*	'twink/chicka'	'A' 'B' 'C' 'D' 'E' 'f' 'G'
		'LF/churr/rattle/jar'	'L'
Yu et al. 2017	*Parus major*	'D'	'L'
Zhang et al. 2022	*Parus minor*	'D'	'L'

Supplementary Table S6. Abbreviations and definitions of acoustic variables calculated for each phrase in the vocal output recorded in response to the experimental presentations of predators.

Variable acronyms	Descriptions
**Duration variables**	
**NS**	Number of syllables in a phrase. This variable characterizes a phrase, i.e., it has one value per phrase.
**PD**	Phrase duration (sec). This variable characterizes a phrase, i.e., it has one value per phrase.
**ASD**	Average from all syllable durations within a phrase (sec). This variable characterizes a phrase, i.e., it has one value per phrase.
**SDSD**	Standard deviation of syllable durations in a phrase (sec); one value per phrase
**ACR**	Average “instantaneous” calling rate (number of syllables/secs in a phrase) calculated as an average from values determined for each syllable in a phrase according to the formula: 1/(preceding inter-syllable interval + syllable duration); one value per phrase
**SDCR**	Standard deviation of the “instantaneous” calling rate (number of syllables/secs in a phrase) calculated for the within-phrase distribution of values for each syllable in a phrase calculated according to the formula: 1/(preceding inter-syllable interval + syllable duration. This variable characterizes a phrase, i.e., it has one value per phrase.
**AISI**	Average from all inter-syllable intervals in a phrase (sec).
**Frequency variables**	
**ACFS**	Average center frequency of syllables in a phrase (Hz) calculated from all center frequencies determined for each syllable in a phrase.
**SDCFS**	Standard deviation of syllable’s center frequency in a phrase (Hz).
**A1QFS**	Average 1st quartile frequency of syllables in a phrase (Hz).
**A3QFS**	Average 3rd quartile frequency of syllables in a phrase (Hz).
**AIRFS**	Average interquartile range frequency of syllables in a phrase (Hz).
**SDIRFS**	Standard deviation from the distribution of interquartile range frequency of syllables in a phrase (Hz).

Supplementary Table S7. Basic data structure for the analyses of detailed acoustic properties of phrases made of Oriental tits' 'L' and Varied tits' 'f' syllables: number of recordings (one recording per nest) and number of phrases recorded per recording in each of the different predator and species group. A brief version of this table is in Table 4 of the main text.

			unit: number of 'L' phrases / recording			
**Paridae species**	**Predator group (Recording Equipment)**	**Number of recordings**	**Mean**	**Standard deviation**	**Min**	**Max**
**Oriental tit**	Plastic snake (Primo EM172, Tascam DR-05)	10	32.2	12.2	8	49
	Live snake (Sennheiser me67, Marantz pmd661 mk2)	9	26	10.6	7	45
	Toy cat (Primo EM172, Tascam DR-05)	8	24.5	20.0	1	56
	Stuffed jay (Sennheiser me67, Tascam DR-100)	8	28.6	10.0	10	43
			**unit: number of 'f' phrases / recording**			
**Varied tit**	Plastic snake (Primo EM172, Tascam DR-05)	11	26.1	11.2	5	44
	Toy cat (Primo EM172, Tascam DR-05)	7	0	0	0	0
	Stuffed jay (Primo EM172, Tascam DR-05)	5	0	0	0	0
